# Supplementary material for: Sas-Ptp10D shapes germ-line stem cell niche by facilitating JNK-mediated apoptosis
Source: PLoS Genet. 2023 Mar 27;19(3):e1010684. doi: 10.1371/journal.pgen.1010684 (PMC10079222; doi:10.1371/journal.pgen.1010684)
Supplement: S1 Fig — (PDF) [file pgen.1010684.s003.pdf]

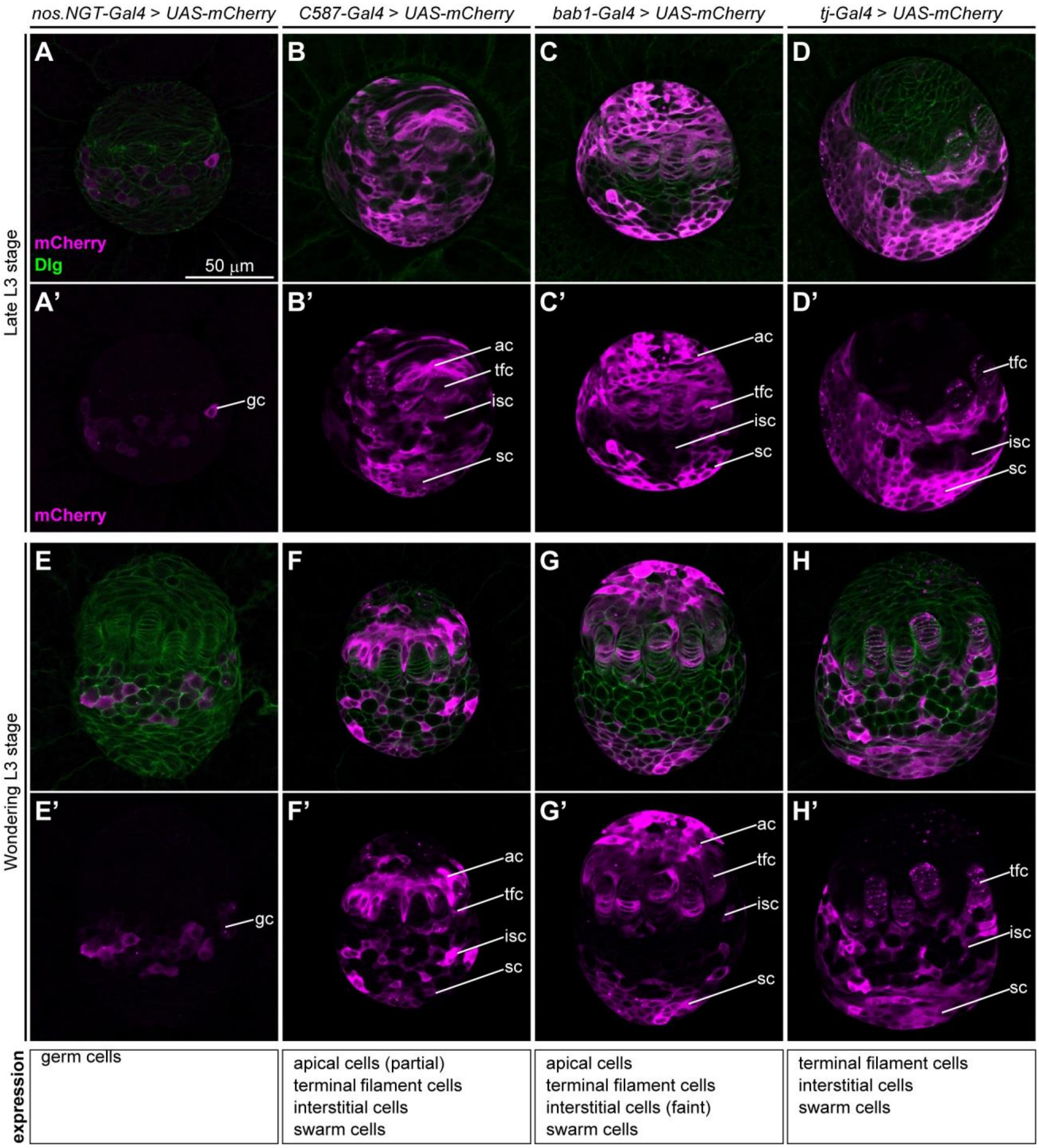

S1 Fig. *Gal4*-expression patterns of *Gal4*-drivers used in the experiments.

(A-D) Female gonads of late L3 larvae (A-D) and wandering larvae (E-H) labeled with mCherry fluorescence (magenta) and anti-Dlg antibody (green). The cell layer located at a opposite side of fat body adherent surface are shown. The expression pattern of *UAS-mCherry* driven by *nos.NGT-Gal4* (A and E), *c587-Gal4* (B), *bab1-Gal4* (C), *tj-Gal*4 (D) are shown. (A'-D') Magenta channels of (A-D). Scale bar in (A) is 50 μm, and applicable for (B-D). The expression patter of each Gal4-driver is indicated at the bottom. gc: germ cells, ac: apical cells, tfc: terminal filament cells, isc: interstitial cells, sc: swarm cells.
