# Supplementary material for: Sas-Ptp10D shapes germ-line stem cell niche by facilitating JNK-mediated apoptosis
Source: PLoS Genet. 2023 Mar 27;19(3):e1010684. doi: 10.1371/journal.pgen.1010684 (PMC10079222; doi:10.1371/journal.pgen.1010684)
Supplement: S2 Fig — (PDF) [file pgen.1010684.s004.pdf]

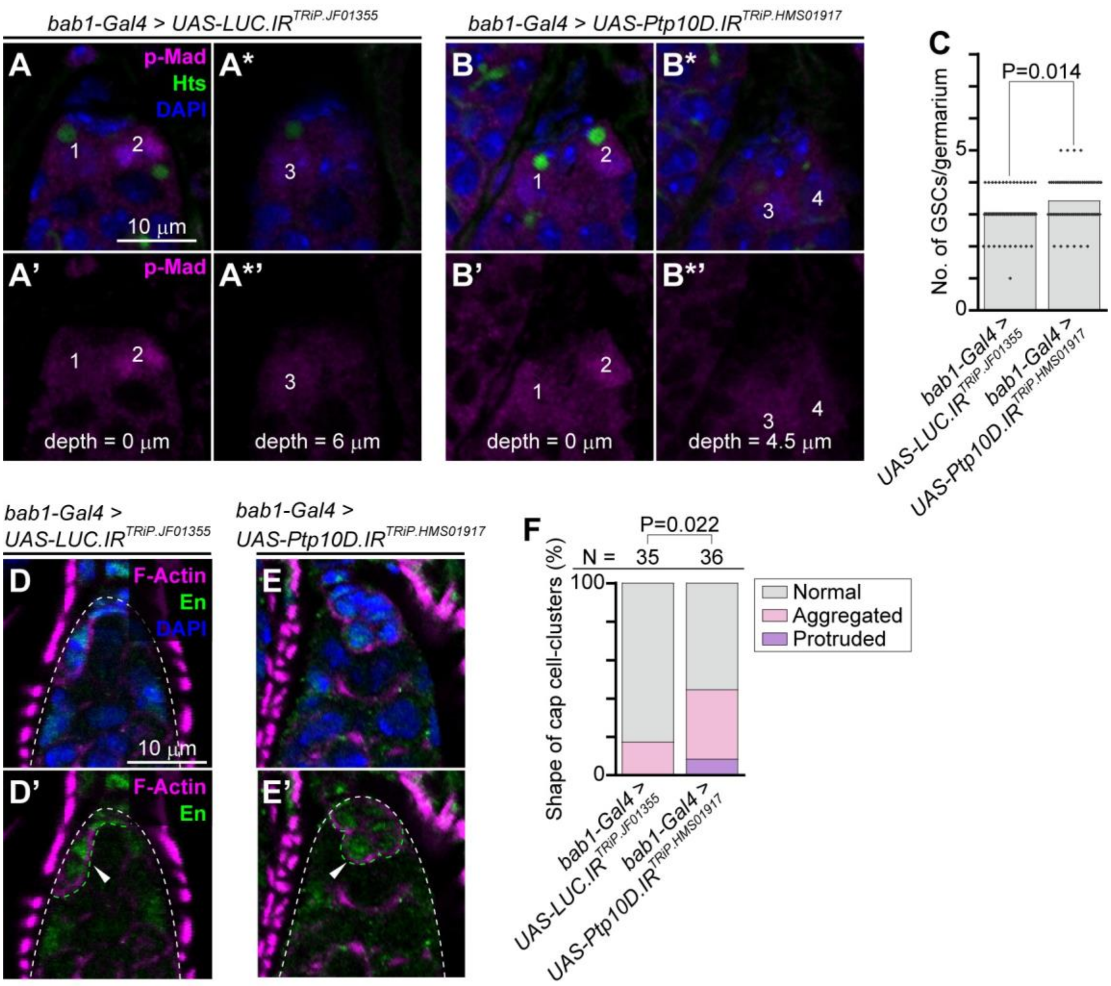

S2 Fig. Germarium phenotype by another *Ptp10D* RNAi line.

(A and B) Proximal regions of female germaria 1 day after eclosion without mating labeled with anti-p-Mad antibody (magenta), anti-Hts antibody (green), and DAPI (blue). Proximal is to the top. Germaria from flies where control *LUC.IR*$^{TRiP.JF01355}$ (A) or *Ptp10D.IR*$^{TRiP.HMS01917}$ (B) are driven by *bab1-Gal4* are shown. Germaria with three (A) and four GSCs (B) are shown as examples. Numberings on (A, A', A*, A*', B, B', B*, and B*') indicate residential GSCs. (A* and B*) Another optical section of (A and B). (A', A*', B', and B*') Magenta channels of (A, A*, B, and B*). Scale bar in (A) is 10 μm, and applicable for (A*, B and B*). (C) Bar graph overlaid with beeswarm plots represents numbers (No.) of GSCs per germarium in indicated genotypes (1 day after eclosion without mating). P-value for Wilcoxon rank sum test is shown at upper. (D and E) Proximal regions of female germaria 1 day after eclosion without mating labeled with Phalloidin (magenta), anti-En antibody (green), and DAPI (blue). Proximal is to the top. Germaria from flies where *LUC.IR*$^{TRiP.JF01355}$ (A) or *Ptp10D.IR*$^{TRiP.HMS01917}$ (B) are driven by *bab1-Gal4* are shown. (D' and E') Magenta/green channels of (D and E). Arrowheads in (D' and E') indicate normal and abnormal cap cell-cluster as examples. Dashed lines in (D' and E') indicate the outlines of germarium. (F) 100%-stacked bar graph

represents % of germaria with normal (gray), aggregated (pink), and burrowing (purple) cap cell-clusters. Numbers (N) of samples observed and P-value for Fisher's exact test are shown at the upper.
