# Supplementary material for: Sas-Ptp10D shapes germ-line stem cell niche by facilitating JNK-mediated apoptosis
Source: PLoS Genet. 2023 Mar 27;19(3):e1010684. doi: 10.1371/journal.pgen.1010684 (PMC10079222; doi:10.1371/journal.pgen.1010684)
Supplement: S3 Fig — (PDF) [file pgen.1010684.s005.pdf]

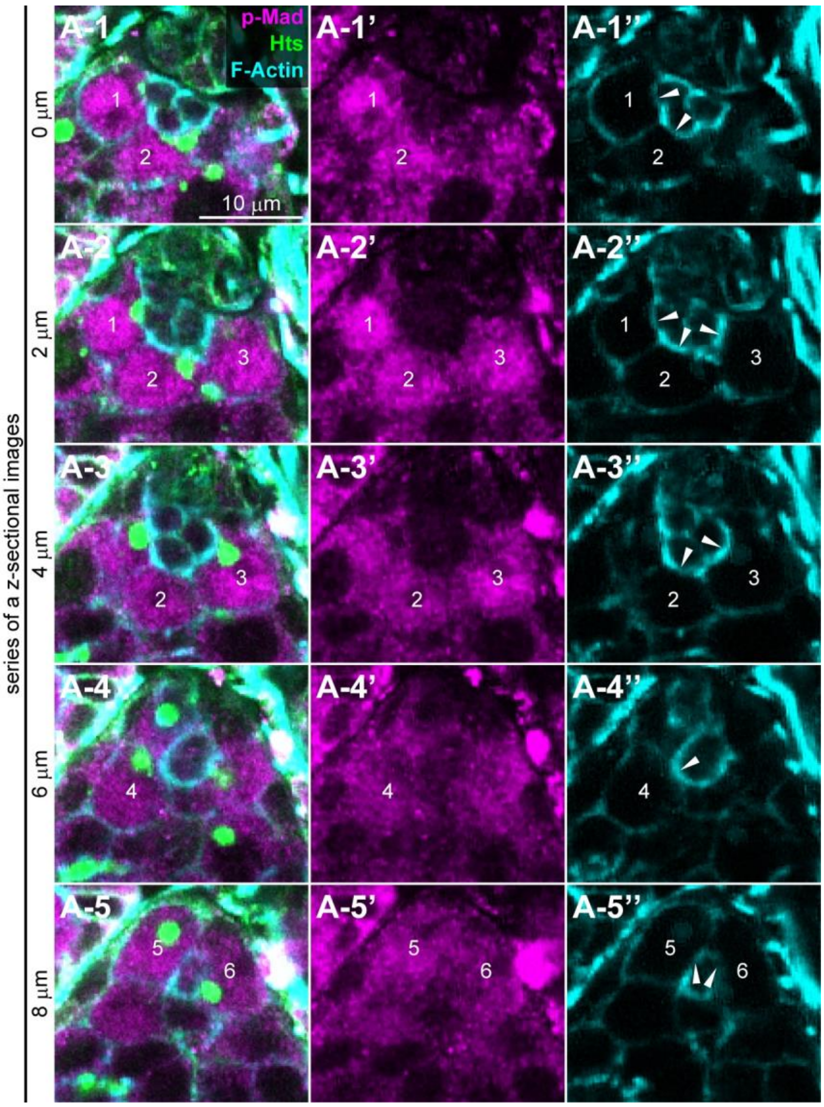

S3 Fig. Excessive GSCs contact with the abnormal cap cell-cluster in *Ptp10D*^{1}.

(A1-A5) Series of optical-sectional images of a germarium in *Ptp10D*^{1} 1 day after eclosion without mating exhibiting the typical abnormal shape of cap cell-cluster with excessive GSCs are labeled with anti-p-Mad antibody (magenta), anti-Hts antibody for spectrosome (green, large dots), and phalloidin for F-Actin (cyan). Depths of confocal sections from (A-1) section are indicated at the left of each image. Six GSCs reside in the germarium are indicated by numbering on the image (1-6) White arrowheads in (A-1''-A-5'') indicate contact sites of GSCs to cap cell-cluster, the outlines of which are distinguishable by the accumulation of F-Actin. (A-1'-A-5') Magenta channels of (A1-A5). (A-1''-A-5'') Cyan channels of (A1-A5). Scale bar in (A) is 10 μm, and applicable for (A-2-A-5).
