# Supplementary material for: Sas-Ptp10D shapes germ-line stem cell niche by facilitating JNK-mediated apoptosis
Source: PLoS Genet. 2023 Mar 27;19(3):e1010684. doi: 10.1371/journal.pgen.1010684 (PMC10079222; doi:10.1371/journal.pgen.1010684)
Supplement: S4 Fig — (PDF) [file pgen.1010684.s006.pdf]

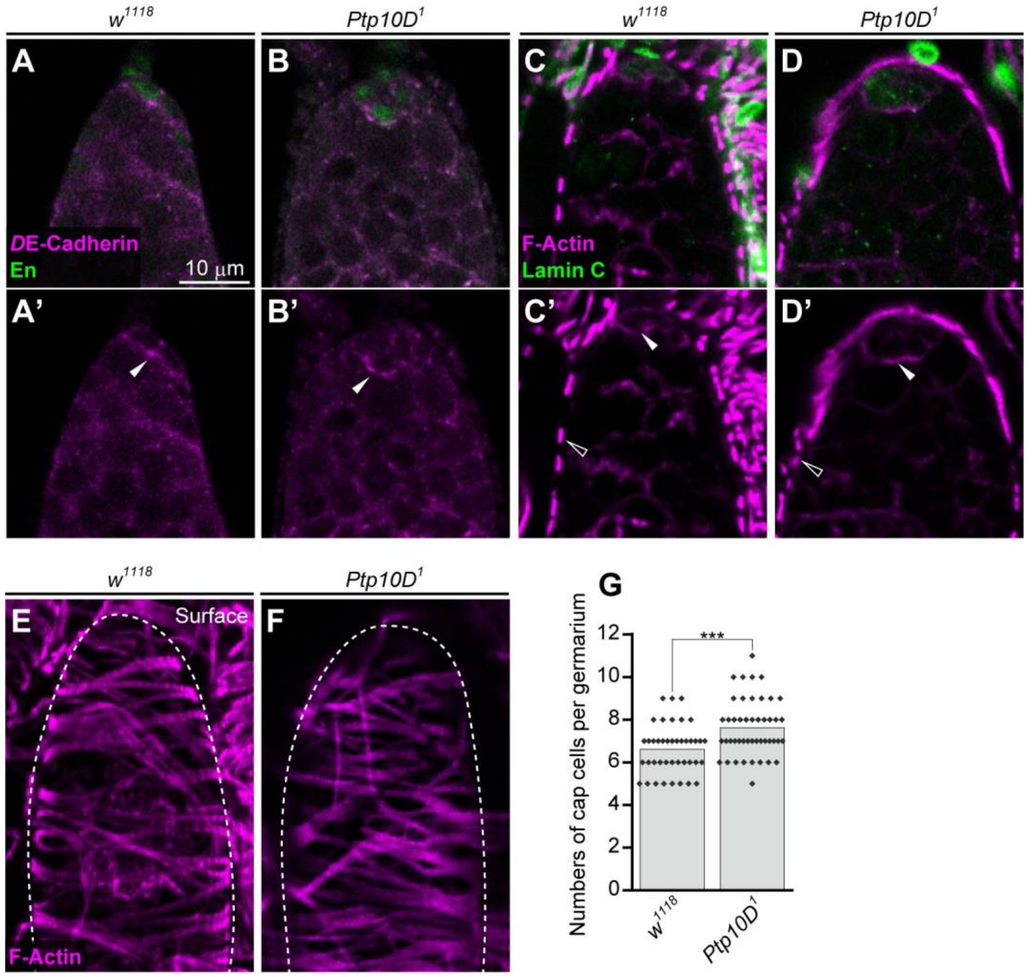

S4 Fig. Morphologies of cap cells and surrounding muscles in *Ptp10D¹*.

(A-E) Proximal regions of female germaria 1 day after eclosion without mating are shown. Germaria are labeled with anti-DE-Cadherin (magenta) and anti-En antibody (green) (A and B), phalloidin (magenta) and anti-Lamin C (green) (C and D), and phalloidin (magenta) (E and F). Proximal is to the top. Germaria from w^{1118} (A, C, and E) and $Ptp10D^1$ (C, D, and F) are shown. (A'-D') Magenta channels of (A-D). (E and F) Confocal sections of basal surfaces corresponding to surrounding muscle sheath are shown. White dashed lines in (E and F) indicate outlines of germaria. Scale bar in (B) is 10 μm, and applicable for (B-F). $Ptp10D^1$ did not exhibit alterations of cap cells in differentiation (En and Lamin C in B and D). cell adhesion (*D*E-Cadherin in B), and actin cytoskeleton (D). The arrangement of muscle sheath was not disrupted in $Ptp10D^1$ (F). (G) Bar graph overlaid with beeswarm plots represent numbers of cap cells per germarium. Genotypes are indicated at the bottom. P-values (***P < 0.0001) for Mann-Whitney U test is shown.
