# Supplementary material for: Sas-Ptp10D shapes germ-line stem cell niche by facilitating JNK-mediated apoptosis
Source: PLoS Genet. 2023 Mar 27;19(3):e1010684. doi: 10.1371/journal.pgen.1010684 (PMC10079222; doi:10.1371/journal.pgen.1010684)
Supplement: S5 Fig — (PDF) [file pgen.1010684.s007.pdf]

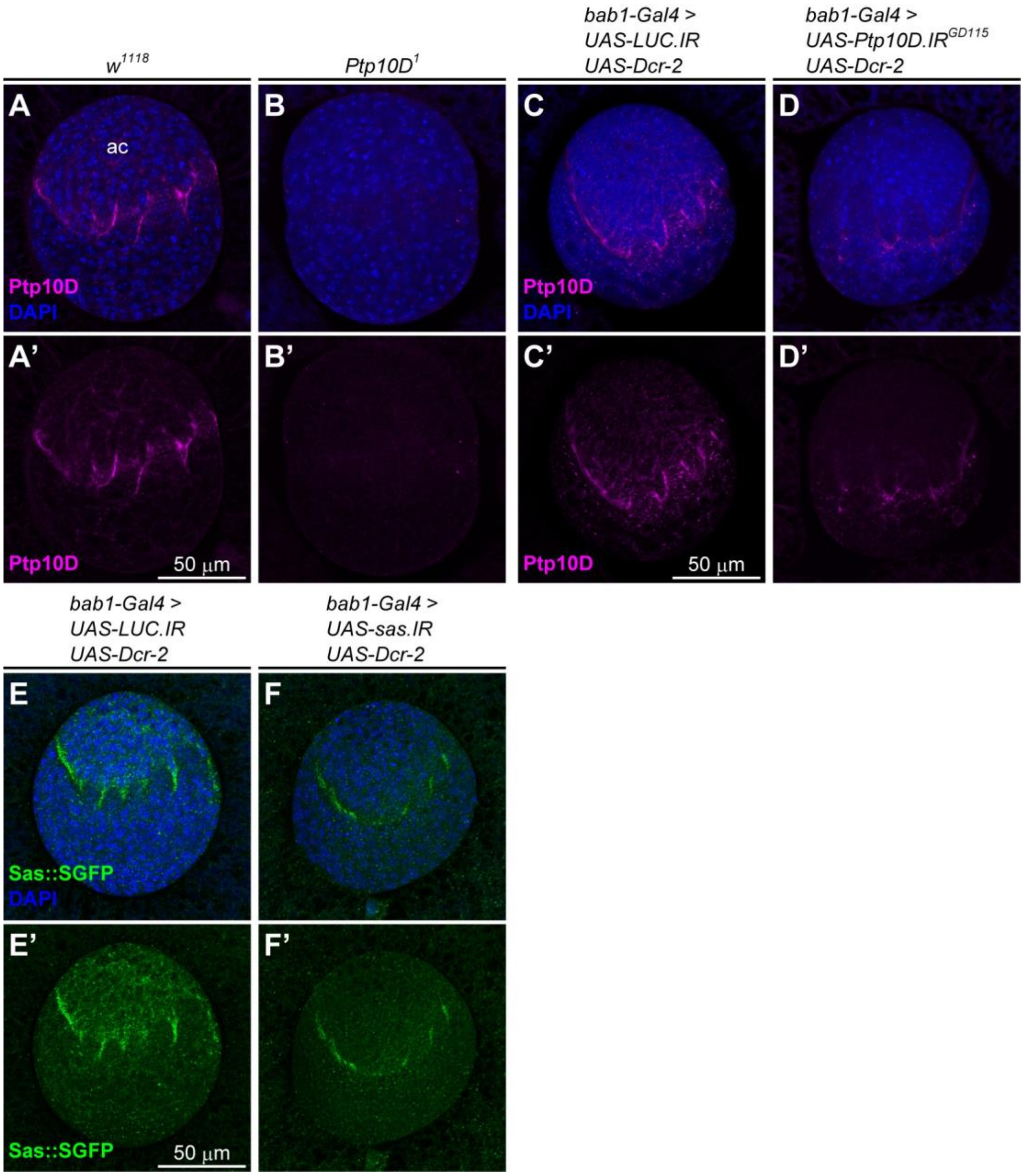

S5 Fig. Validation of antibodies and RNAi lines.

(A-D) Female gonads of indicated genotypes (upper) at wondering L3 stages are labeled with DAPI (blue) and anti-Ptp10D antibody (magenta) (A-D), and anti-GFP antibody for Sas::SGFP (green) (E and F) are shown. The cell layer located at the opposite side of the fat body adherent surface are shown. Images are processed by the Z-stack projection of two sections (corresponding to 3 µm thickness). (A'-D') Magenta channels of (A-D). (E' and F') Green channels of (E and F). Scale bars, 50 µm. *Ptp10D*^{1} exhibited a complete loss of Ptp10D staining in gonad (A and B). Knockdown of *Ptp10D* by *bab1-Gal4* reduced the Ptp10D staining especially in apical cells (C and D). Knockdown of *sas* by *bab1-Gal4* reduced Sas::SGFP (E and F) in apical cells.
