# Supplementary material for: Sas-Ptp10D shapes germ-line stem cell niche by facilitating JNK-mediated apoptosis
Source: PLoS Genet. 2023 Mar 27;19(3):e1010684. doi: 10.1371/journal.pgen.1010684 (PMC10079222; doi:10.1371/journal.pgen.1010684)
Supplement: S6 Fig — (PDF) [file pgen.1010684.s008.pdf]

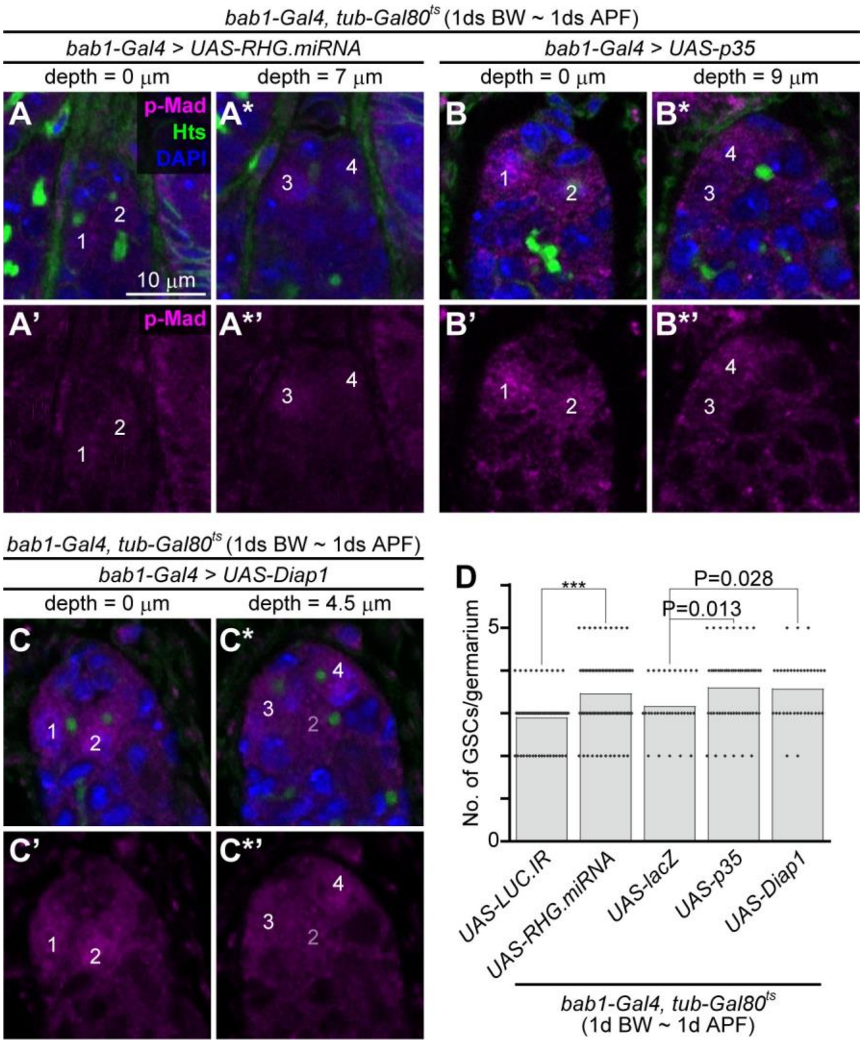

S6 Fig. Blocking apoptosis causes increased number of GSCs.

(A-C) Proximal regions of virgin female germaria labeled with anti-p-Mad antibody (magenta), anti-Hts antibody (green), and DAPI (blue). Proximal is to the top. Germaria of indicated genotypes 2 days after eclosion that are treated with temperature-sift (29°C, 1d BW ~ 1d APF) are shown. Germaria with four GSCs are shown as examples. Numberings indicate residential GSCs. (A*-C*) Another optical section of (A-C). (A'-C', and A*'-C*') Magenta channels of (A-C and A*-C*). Scale bar in (A) is 10 μm, and applicable for (B, C, and A*-C*). (C) Bar graph overlaid with beeswarm plots represents numbers (No.) of GSCs per germarium in indicated genotypes. P-values (***P < 0.0001, *P<0.01, 0.01 < "actual P-value" < 0.05) for Mann-Whitney U test are shown.
