# Supplementary material for: Sas-Ptp10D shapes germ-line stem cell niche by facilitating JNK-mediated apoptosis
Source: PLoS Genet. 2023 Mar 27;19(3):e1010684. doi: 10.1371/journal.pgen.1010684 (PMC10079222; doi:10.1371/journal.pgen.1010684)
Supplement: S7 Fig — (PDF) [file pgen.1010684.s009.pdf]

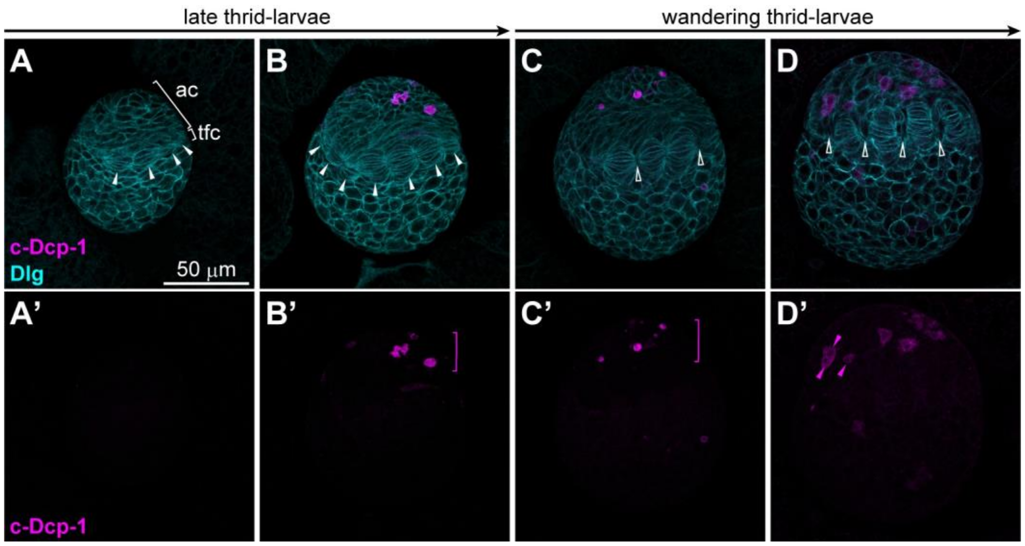

S7 Fig. Apoptosis in apical cells during larval gonadogenesis.

(A-D) Female gonads at indicated developmental stages are labeled with anti-c-Dcp-1 antibody (magenta) and anti-Dlg antibody (cyan). The cell layer located at a opposite side of fat body adherent surface are shown. Images of gonads at late-third larval stage (A and B) and wandering-third larval stage (C and D) are arranged from the left in the order of developmental stages. Images are processed by the Z-stack projection of two sections (corresponding to 3 μm thickness) to visualize the outlines of terminal filaments. (A'-D') Magenta channels of (A-D). Scale bar in (A) is 50 μm, and applicable for (B-D and A'-D'). White brackets in (A) indicate the region of apical cells (ac) and terminal filament cells (tfc). White arrowheads in (A and B) indicate rows of terminal filaments. White open arrowheads indicate the apical cells which separate rows of terminal filament cells. Magenta brackets in (B' and C') indicate apoptosis-emerging region. Magenta arrowheads in (D') indicate apoptosis in apical cells adjacent to terminal filament cells.
