# Supplementary material for: Sas-Ptp10D shapes germ-line stem cell niche by facilitating JNK-mediated apoptosis
Source: PLoS Genet. 2023 Mar 27;19(3):e1010684. doi: 10.1371/journal.pgen.1010684 (PMC10079222; doi:10.1371/journal.pgen.1010684)
Supplement: S8 Fig — (PDF) [file pgen.1010684.s010.pdf]

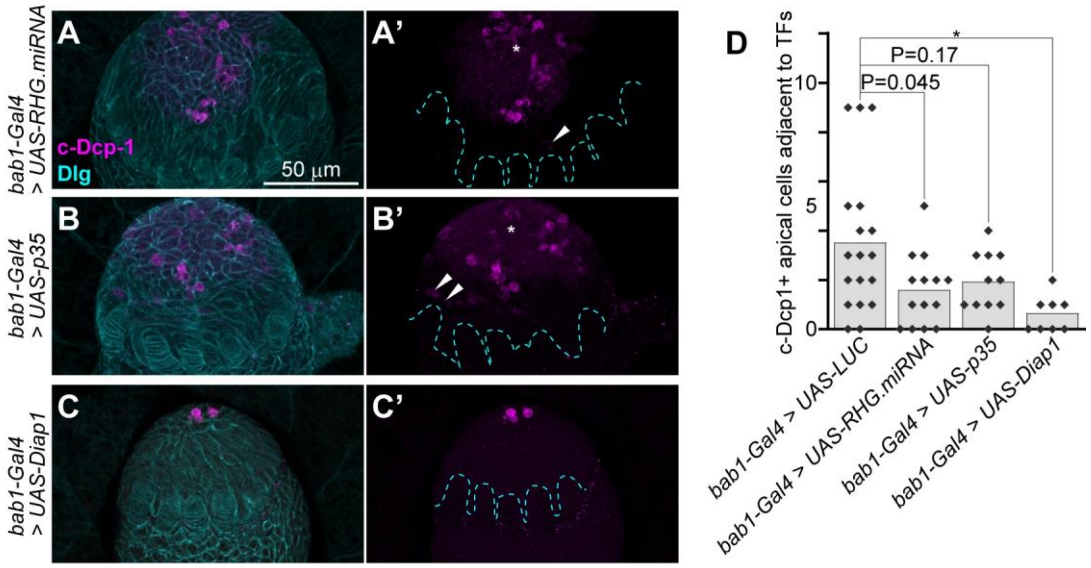

S8 Fig. Overexpression of apoptosis inhibitors suppress apoptosis in apical cells.

(A-D) Female gonads at wondering L3 stage are labeled with anti-c-Dcp-1 antibody (magenta) and anti-Dlg antibody (cyan). The cell layer located at the opposite side of the fat body adherent surface are shown. Genotypes of samples are indicated at the left. Images are processed by Z-stack projection of two sections (corresponding to 3 μm thickness) to visualize the boundary between apical cells and terminal filament cells. (A'-C') Magenta channels of (A-C). Scale bars, 50 μm. Cyan dashed lines in (A'-C') indicate boundary between apical cells and terminal filament cells. Arrowheads in (A' and B') indicate the c-Dcp-1-positive apical cells which are adjacent to terminal filaments. Asterisks in A' and B' indicate accumulation of c-Dcp-1 positive cells at the proximal regions by unknown mechanism. (G) Bar graph overlaid with beeswarm plots represent numbers (No.) of c-Dcp-1-positive apical cells adjacent to terminal filaments (TFs) per larval gonads in indicated genotypes. *LUC* was used as control. P-values ($*P < 0.01$, $0.01 <$ "actual P-value") for Mann-Whitney U test are shown.
