# Supplementary material for: Sas-Ptp10D shapes germ-line stem cell niche by facilitating JNK-mediated apoptosis
Source: PLoS Genet. 2023 Mar 27;19(3):e1010684. doi: 10.1371/journal.pgen.1010684 (PMC10079222; doi:10.1371/journal.pgen.1010684)
Supplement: S9 Fig — (PDF) [file pgen.1010684.s011.pdf]

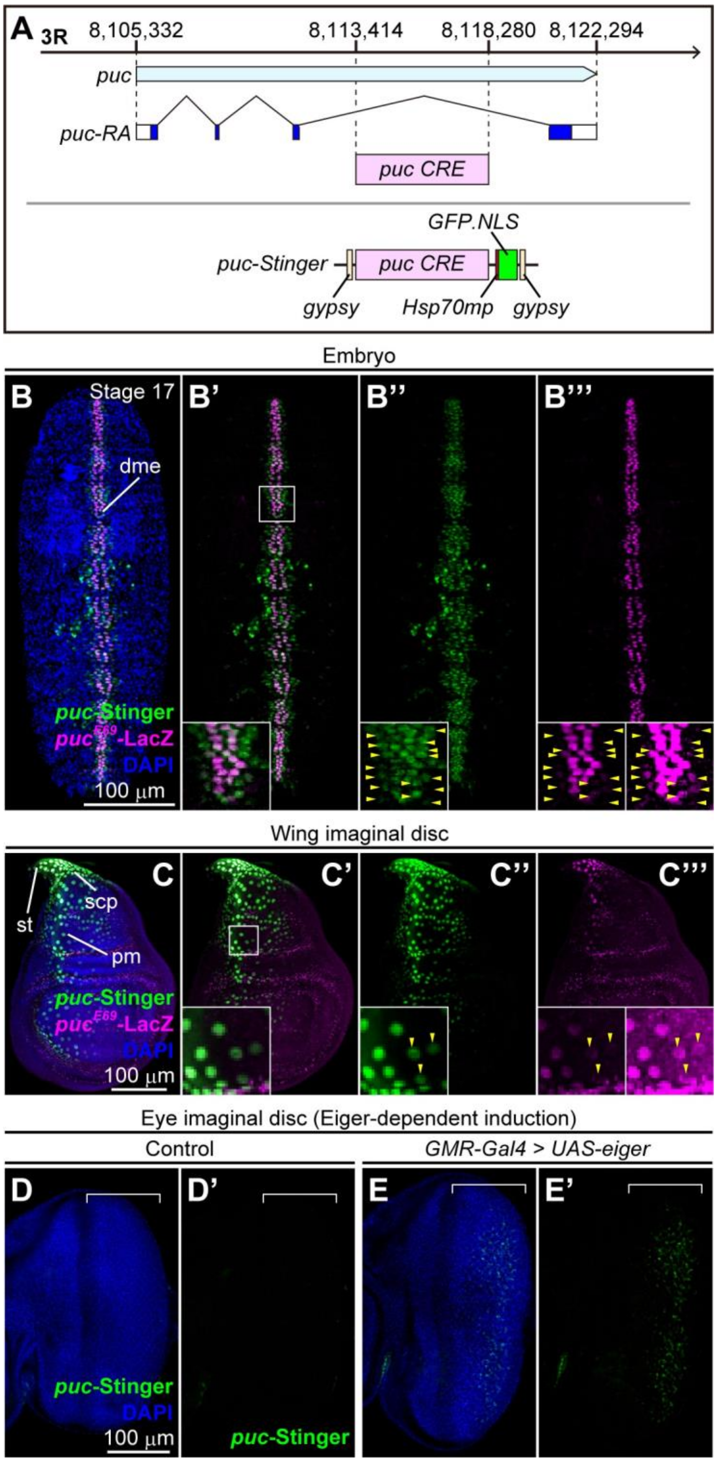

S9 Fig. Validation of *puc*-Stinger, a high-sensitive reporter of JNK signaling.

(A) Cytological map around *puc* gene region (upper) and the structure of *puc-Stinger* construct (lower). Pink box indicates the location including a JNK-responsible element which was subcloned into *pH-Stinger* plasmid for the construction of *puc-Stinger*. (B and D) Comparison of expression pattern between *puc*-Stinger and *puc*^{E69}*-lacZ*, a well-established *puc* reporter. Images processed by the Z-stack projection are shown. (B) Dorsal views of embryos at stage17 with one of *puc-Stinger* transgenes

(green) and *puc*^{E69}*-lacZ* is labelled with anti-β-Gal antibody (magenta) and DAPI (blue). Anterior is to the top. dme: dorsal most epidermal cells. (B', B", and B"') Green/magenta, green, and magenta channels of (B), respectively. Scale bar in (B) is 100 μm, and applicable for (B'-B"'). (C) Wing imaginal disc of wandering L3 larva with one of *puc-Stinger* transgenes (green) and *puc*^{E69}*-lacZ* is labelled with anti-β-Gal antibody (magenta) and DAPI (blue). Anterior and dorsal are to the left and top, respectively. st: stalk cells, scp: scutellum primordial cells. (C', C", and C"') Green/magenta, green, and magenta channels of (C), respectively. Magnified images of white bracket in (B' and C') are shown at left-lower insets. Right-lower insets in (B"' and C"') are high-intensity image of left-lower insets in (B"' and C"') created by the digital processing. *puc*-Stinger exhibits clear signal intensities in cells with faint *puc*^{E69}*-lacZ* signals (yellow arrowheads in insets of B", B"', C" and C"). (D and E) Eiger/TNFα-dependent induction of *puc-Stinger*. Eye imaginal discs of wandering L3 larva with one copy of *puc-Stinger* transgene (green) are labelled with DAPI (blue). Genotypes are indicated at the top. Anterior and dorsal are to the left and top, respectively. Scale bar in (D) is 100 μm, and applicable for (E). (D' and E') Green channels of (D and E). Overexpression of *eiger* driven by *GMR-Gal4* induced the expression of *puc-Stinger* at photoreceptor region corresponding to the *GMR* enhancer-activated region (white brackets in E and E' compared with D and D').
