# Supplementary material for: Sas-Ptp10D shapes germ-line stem cell niche by facilitating JNK-mediated apoptosis
Source: PLoS Genet. 2023 Mar 27;19(3):e1010684. doi: 10.1371/journal.pgen.1010684 (PMC10079222; doi:10.1371/journal.pgen.1010684)
Supplement: S10 Fig — (PDF) [file pgen.1010684.s012.pdf]

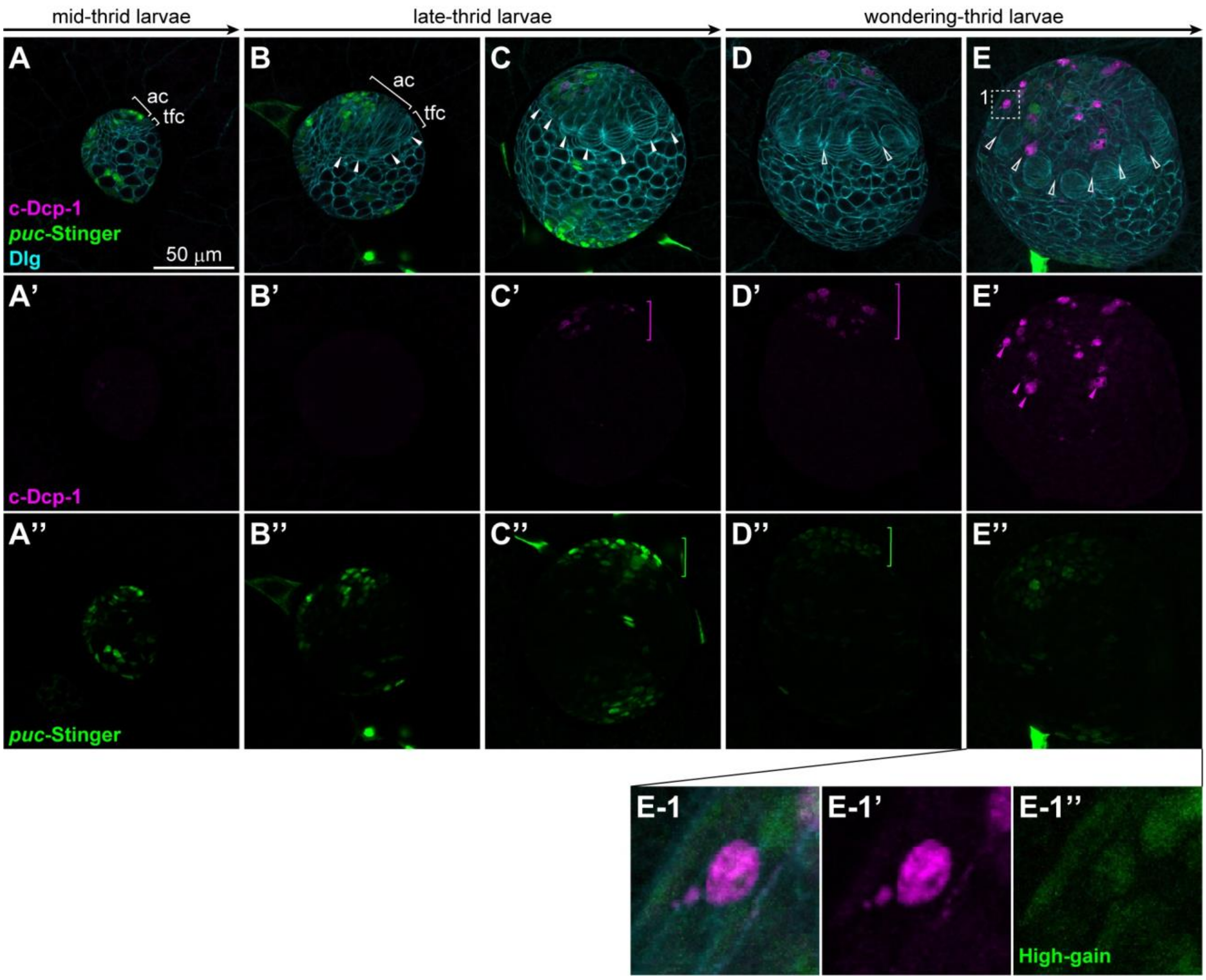


**S10 Fig. Apoptosis and JNK signaling activity in gonadal apical cells.**

(A-E) Female gonads at indicated developmental stages are labeled with anti-c-Dcp-1 antibody (magenta), *puc*-Stinger fluorescence (green), and anti-Dlg antibody (cyan). The cell layer located at a opposite side of fat body adherent surface are shown. Images of gonads at mid-third larval stage (A), late-third larval stage (B and C) and wandering-third larval stage (D and E) are arranged from the left in the order of developmental stages. Images are processed by the Z-stack projection of two sections (corresponding to 3 μm thickness) to visualize the outlines of terminal filaments. (A'-E') Magenta channels of (A-E). (A''-E'') Green channels of (A-E). Scale bar in (A) is 50 μm, and applicable for (B-E, A'-E' and A''-E''). White brackets in (A and B) indicate the region of apical cells (ac) and terminal filament cells (tfc). White arrowheads in (B and C) indicate rows of terminal filaments. White open arrowheads indicate apical cells which separate rows of terminal filament cells. Magenta brackets in (C and D) indicate apoptosis-emerging region. Magenta arrowheads in (E') indicate apoptosis in apical cells adjacent to terminal filament cells. Green brackets in (C'' and D'') indicate JNK signaling-activated region. (E-1-E-1'') High-magnification images of white-dashed box 1 shown in E. Gains of

green channels are amplified to enhance the signal. Some apical cells adjacent to terminal filament cells show *puc*-Stinger signal (E-1’’) and undergo apoptosis (E-1’).
