# Supplementary material for: Sas-Ptp10D shapes germ-line stem cell niche by facilitating JNK-mediated apoptosis
Source: PLoS Genet. 2023 Mar 27;19(3):e1010684. doi: 10.1371/journal.pgen.1010684 (PMC10079222; doi:10.1371/journal.pgen.1010684)
Supplement: S11 Fig — (PDF) [file pgen.1010684.s013.pdf]

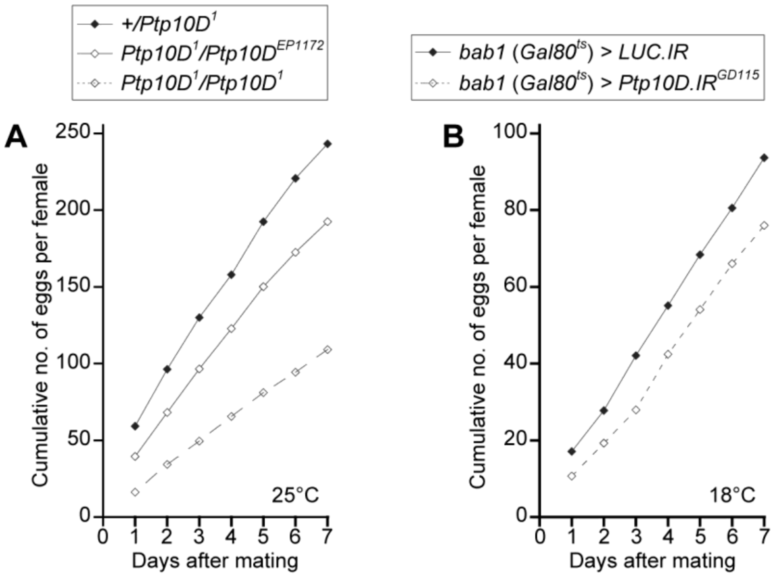

S11 Fig. Loss of Ptp10D diminished the egg production.

(A and B) Line graphs represent means of cumulative numbers of eggs laid by single females after mating with single wild-type *Canton-S* males. (A) Data values from control *+/Ptp10D*^{1} (black line and closed dots in A), *Ptp10D*^{1}*/Ptp10D*^{EP1172} (black line and open dots in A) and *Ptp10D*^{1}*/Ptp10D*^{1} (dashed line and open dots in A) at 25 degrees. (B) Data values from control *UAS-LUC.IR* (black line and closed dots in B) and *UAS-Ptp10D.IR* (dashed line and open dots in B) by *bab1-Gal4*/TARGET at 18 degrees. (A' and B').
