# Supplementary material for: Sas-Ptp10D shapes germ-line stem cell niche by facilitating JNK-mediated apoptosis
Source: PLoS Genet. 2023 Mar 27;19(3):e1010684. doi: 10.1371/journal.pgen.1010684 (PMC10079222; doi:10.1371/journal.pgen.1010684)
Supplement: S12 Fig — (PDF) [file pgen.1010684.s014.pdf]

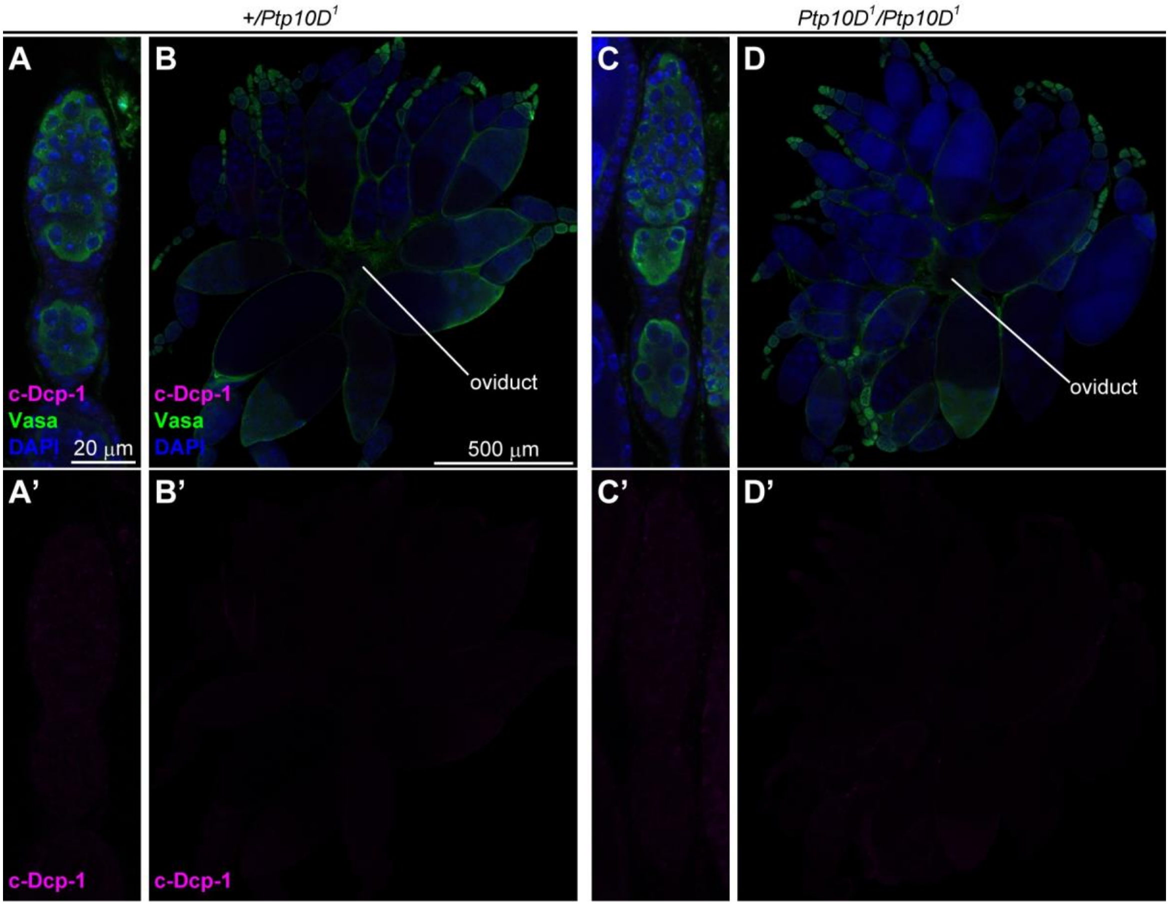

S12 Fig. *Ptp10D*^{1} does not exhibit increased apoptosis in oogenesis.

(A-D) Virgin female germaria (A and C) and ovaries (B and D) loosened radially 1 day after eclosion are labeled with anti-c-Dcp-1 antibody (magenta), anti-Vasa antibody (green), and DAPI (blue). Control *Ptp10D*^{1} heterozygote (A and B) and *Ptp10D*^{1} homozygote (C and D) are shown. Scale bars, 20 μm (A and C) or 500 μm (B and D). *Ptp10D*^{1} homozygote did not exhibit increase in the number of apoptotic cells in germarium (C) or oocytes (D).
