# Supplementary material for: Sas-Ptp10D shapes germ-line stem cell niche by facilitating JNK-mediated apoptosis
Source: PLoS Genet. 2023 Mar 27;19(3):e1010684. doi: 10.1371/journal.pgen.1010684 (PMC10079222; doi:10.1371/journal.pgen.1010684)
Supplement: S1 File — (DOCX) [file pgen.1010684.s015.docx]

**S1 File. Genotypes of flies used in the experiments.**

**Fig 1**

(B, B’) *w^1118^/w^1118^; +/+; +/+*

(C, C*, C’, C*’) *w*, Ptp10D^1^/w*, Ptp10D^1^; +/+; +/+*

(D, lane 1) *w^1118^/w^1118^; +/+; +/+*

(D, lane 2) *w*, Ptp10D^1^/w*, Ptp10D^1^; +/+; +/+*

(D, lane 3) *w*/ w^1118^; Egfr^f2^/+; +/+*

(D, lane 4) *w*, Ptp10D^1^/ w^1118^, Ptp10D^EP1172^; Egfr^f2^/+; +/+*

(D, lane 5) *w*, Ptp10D^1^/w^1118^, Df(1)Exel6242; +/+; +/+*

(D, lane 6) *w*, Ptp10D^1^/w^1118^, Ptp10D^EP1172^; +/+; +/+*

(D, lane 7) *w*, Ptp4E^1^/w*, Ptp4E^1^; +/+; +/+*

(E, lane 1) *w*/w*; +/UAS-Dcr-2; +/UAS-Ptp10D.IR^GD115^*

(E, lane 2) *y^1^, w*/w*; nos.NGT-Gal4/UAS-Dcr-2; +/UAS-Ptp10D.IR^GD115^*

(E, lane 3) *C587-Gal4, w*/w*; +/UAS-Dcr-2; +/UAS-Ptp10D.IR^GD115^*

(E, lane 4) *w*/w*; +/UAS-Dcr-2; bab1-Gal4/UAS-Ptp10D.IR^GD115^*

(E, lane 5) *y*, w*/w*; tj-Gal4/UAS-Dcr-2; +/UAS-Ptp10D.IR^GD115^*

(E, lane 6) *w*/w*; +/UAS-sas.IR^GD14869^; +/UAS-Dcr-2*

(E, lane 7) *y^1^, w*/w*; nos.NGT-Gal4/UAS-sas.IR^GD14896^; +/UAS-Dcr-2*

(E, lane 8) *C587-Gal4, w*/w*; +/UAS-sas.IR^GD14869^; +/UAS-Dcr-2*

(E, lane 9) *w*/w*; +/UAS-sas.IR^GD1486^; bab1-Gal4/UAS-Dcr-2*

(E, lane 10) *y*, w*/w*; tj-Gal4/UAS-sas.IR^GD14869^; +/UAS-Dcr-2*

(F, lane 1-4) *w*/w*; tub-Gal80^ts^/UAS-Dcr-2; bab1-Gal4/UAS-Ptp10D.IR^GD115^*

(F, lane 5-8) *w*/w*; tub-Gal80^ts^/UAS-sas.IR^GD14869^; bab1-Gal4/UAS-Dcr-2*

**Fig 2**

(A, A’) *w^1118^/w^1118^; +/+; +/+*

(B, B’, C, C’) *w*, Ptp10D^1^/w*, Ptp10D^1^; +/+; +/+*

(D, D’) *w*/w*; UAS-Dcr-2/tub-Gal80^ts^; UAS-LUC.IR^TRiP.JF01355^/bab1-Gal4*

(E, E’) *w*/w*; UAS-Dcr-2/tub-Gal80^ts^; UAS-Ptp10D.IR^GD115^/bab1-Gal4*

(F, F’) *w*/w*; UAS-sas.IR^GD14869^/tub-Gal80^ts^; UAS-Dcr-2/bab1-Gal4*

(G, lane 1) *w^1118^/w^1118^; +/+; +/+*

(G, lane 2) *w*, Ptp10D^1^/w*, Ptp10D^1^; +/+; +/+*

(G, lane 3) *w*/w*; UAS-Dcr-2/tub-Gal80^ts^; UAS-LUC.IR^TRiP.JF01355^/bab1-Gal4*

(G, lane 4) *w*/w*; UAS-Dcr-2/tub-Gal80^ts^; UAS-Ptp10D.IR^GD115^/bab1-Gal4*

(G, lane 5) *w*/w*; UAS-sas.IR^GD1486^/tub-Gal80^ts^; UAS-Dcr-2/bab1-Gal4*

(H-H’’) *w*/w*; +/+; sas::SGFP/sas::SGFP*

**Fig 3**

(A, A-1, A’, A-1’) *w^1118^/w^1118^; +/+; +/+*

(B, B-1, B’, B-1’) *w*, Ptp10D^1^/w*, Ptp10D^1^; +/+; +/+*

(C, lane 1) *w^1118^/w^1118^; +/+; +/+*

(C, lane 2) *w*, Ptp10D^1^/w*, Ptp10D^1^; +/+; +/+*

(D, lane 1) *w^1118^/w*; UAS-lexA.IR^TRiP.HMS05772^/tub-Gal80^ts^; +/bab1-Gal4*

(D, lane 2) *w*, Ptp10D^1^/ w*, Ptp10D^1^; tub-Gal80^ts^,UAS-lexA.IR^TRiP.HMS05772^/+; +/bab1-Gal4*

(D, lane 3) *w*, Ptp10D^1^/ w*, Ptp10D^1^; tub-Gal80^ts^, UAS-Egfr.IR^10079R-1^/+; +/bab1-Gal4*

(E, E-1, E-2, E’, E-1’, E-2’) *w*/w^1118^; tub-miniCic::mCherry/+; +/+*

(F) *w*/w^1118^; tub-miniCic::mCherry/+; +/+*

**Fig 4**

(A, A’) *w*/w*; UAS-lexA.IR^TRiP.HMS05772^/tub-Gal80^ts^; +/bab1-Gal4*

(B, B) *w*/w*; UAS-RHG.miRNA/tub-Gal80^ts^; +/bab1-Gal4*

(C, lane 1) *w*/w*; UAS-lexA.IR^TRiP.HMS05772^/tub-Gal80^ts^; +/ bab1-Gal4*

(C, lane 2) *w*/w*; UAS-RHG.miRNA/tub-Gal80^ts^; +/bab1-Gal4*

(C, lane 3) *w^1118^/w*; +/tub-Gal80^ts^; UAS-LUC/bab1-Gal42*

(C, lane 4 *w^1118^/w*; UAS-p35/tub-Gal80^ts^; +/bab1-Gal4*

(C, lane 5 *w*, UAS-Diap1/w*; +/tub-Gal80^ts^; +/bab1-Gal4*

(D, D’) *w^1118^/ w^1118^; +/+; +/+*

(E, E’) *w*, Ptp10D^1^/ w*, Ptp10D^1^; +/+; +/+*

(F, F’) *w*, Ptp10D^1^/ w*, Ptp10D^1^; UAS-Egfr.IR^10079R-1^/+; +/bab1-Gal4*

(G, lane 1) *w^1118^/w^1118^; +/+; +/+*

(G, lane 2) *w*, Ptp10D^1^/ w*, Ptp10D^1^; +/+; +/+*

(G, lane 3) *w*, Ptp10D^1^/ w*, Ptp10D^1^; +/+; UAS- LUC.IR^TRiP.JF01355^/bab1-Gal4*

(G, lane 4) *w*, Ptp10D^1^/ w*, Ptp10D^1^; UAS-Egfr.IR^10079R-1^/+; +/bab1-Gal4*

**Fig 5**

(A, A’) *w*/w*; +/tub-Gal80^ts^; UAS-LUC/bab1-Gal4*

(B, B’) *w*/w*; +/tub-Gal80^ts^; UAS-puc/bab1-Gal4*

(C, lane 1) *w*/w*; +/tub-Gal80^ts^; UAS-LUC/bab1-Gal4*

(C, lane 2) *w*/w*; +/tub-Gal80^ts^; UAS-puc/bab1-Gal4*

(D, D’) *w^1118^/ w*; +/+; UAS-puc/bab1-Gal4*

(E, lane 1) *w*/w*; +/+; UAS-LUC/bab1-Gal4*

(E, lane 2) *w^1118^/ w*; +/+; UAS-puc/bab1-Gal4*

(F, F’) *w^1118^/ w^1118^; puc-Stinger/+; +/+*

(G, G’) *w*, Ptp10D^1^/ w*, Ptp10D^1^; puc-Stinger/+; +/+*

(H, H’) *w*, Ptp10D^1^/ w*, Ptp10D^1^; puc-Stinger*, *UAS-Egfr.IR/+; +/bab1-Gal4*

(I, lane 1, 5) *w^1118^/ w^1118^; puc-Stinger/+; +/+*

(I, lane 2, 6) *w*, Ptp10D^1^/ w*, Ptp10D^1^; puc-Stinger/+; +/+*

(I, lane 3, 7) *w*, Ptp10D^1^/ w*, Ptp10D^1^; puc-Stinger*, *UAS-lexA.IR/+; +/bab1-Gal4*

(I, lane 4, 8) *w*, Ptp10D^1^/ w*, Ptp10D^1^; puc-Stinger*, *UAS-Egfr.IR/+; +/bab1-Gal4*

**Fig 6**

(A, black line, closed dots) *w*, Ptp10D^1^/w^1118^; +/+; +/+*

(A, black line, open dots) *w*, Ptp10D^1^/w*, Ptp10D^EP1172^; +/+; +/+*

(A, dashed line, open dots) *w*, Ptp10D^1^/w*, Ptp10D^1^; +/+; +/+*

(A’, lane 1) *w*, Ptp10D^1^/w^1118^; +/+; +/+*

(A’, lane 2) *w*, Ptp10D^1^/w*, Ptp10D^EP1172^; +/+; +/+*

(A’, lane 3) *w*, Ptp10D^1^/w*, Ptp10D^1^; +/+; +/+*

(B, black line, closed dots) *w*/w*; UAS-Dcr-2/tub-Gal80^ts^; UAS-LUC.IR^TRiP.JF01355^/bab1-Gal4*

(B, dashed line, open dots) *w*/w*; UAS-Dcr-2/tub-Gal80^ts^; UAS-Ptp10D.IR^GD115^/bab1-Gal4*

(B’, lane 1) *w*/w*; w*/w*; UAS-Dcr-2/tub-Gal80^ts^; UAS-LUC.IR^TRiP.JF01355^/bab1-Gal4*

(B’, lane 2) *w*/w*; UAS-Dcr-2/tub-Gal80^ts^; UAS-Ptp10D.IR^GD115^/bab1-Gal4*

**S1 Movie**

*w^1118^/ w^1118^; +/+; +/+*

**S2 Movie**

*w*, Ptp10D^1^/ w*, Ptp10D^1^; +/+; +/+*

**S1 Fig**

(A, A’, E, E’) *y^1^, w*/w*; nos.NGT-Gal4/+; +/UAS-mCherry*

(B, B’, F, F’) *C587-Gal4, w*/w*; +/+; +/UAS-mCherry*

(C, C’, G, G’) *y*, w*/w*; +/+; bab1-Gal4/UAS-mCherry*

(D, D’, H, H’) *y*, w*/w*; tj-Gal4/+; +/UAS-mCherry*

**S2 Fig**

(A, A’, A*, A*) *w*/w*; +/+; UAS-LUC.IR^TRiP.JF01355^/bab1-Gal4*

(B, B’, B*, B*) *w*/w*; +/+; UAS-Ptp10D.IR^TRiP.HMS01917^/bab1-Gal4*

(C, lane1) *w*/w*; +/+; UAS-LUC.IR^TRiP.JF01355^/bab1-Gal4*

(C, lane2) *w*/w*; +/+; UAS-Ptp10D.IR^TRiP.HMS01917^/bab1-Gal4*

(D, D’) *w*/w*; +/+; UAS-LUC.IR^TRiP.JF01355^/bab1-Gal4*

(E, E’) *w*/w*; +/+; UAS-Ptp10D.IR^TRiP.HMS01917^/bab1-Gal4*

(F, lane1) *w*/w*; +/+; UAS-LUC.IR^TRiP.JF01355^/bab1-Gal4*

(F, lane2) *w*/w*; +/+; UAS-Ptp10D.IR^TRiP.HMS01917^/bab1-Gal4*

**S3 Fig**

*w*, Ptp10D^1^/w*, Ptp10D^1^; +/+; +/+*

**S4 Fig**

(A, A’) *w^1118^/ w^1118^; +/+; +/+*

(B, B’) *w*, Ptp10D^1^/ w*, Ptp10D^1^; +/+; +/+*

(C, C’) *w^1118^/ w^1118^; +/+; +/+*

(D, D’) *w*, Ptp10D^1^/ w*, Ptp10D^1^; +/+; +/+*

(E, E’) *w^1118^/ w^1118^; +/+; +/+*

(F, F’) *w*, Ptp10D^1^/ w*, Ptp10D^1^; +/+; +/+*

(G, lane 1) *w^1118^/ w^1118^; +/+; +/+*

(G, lane 2) *w*, Ptp10D^1^/ w*, Ptp10D^1^; +/+; +/+*

**S5 Fig**

(A, A’) *w^1118^/ w^1118^; +/+; +/+*

(B, B’) *w*, Ptp10D^1^/ w*, Ptp10D^1^; +/+; +/+*

(C, C’) *w*/w*; +/UAS-Dcr-2; bab1-Gal4/UAS-LUC.IR^TRiP.JF01355^*

(D, D’) *w*/w*; +/UAS-Dcr-2; bab1-Gal4/UAS-Ptp10D.IR^GD115^*

(E, E’) *w*/w*; +/UAS-Dcr-2; bab1-Gal4, sas::SGFP/UAS-LUC.IR^TRiP.JF01355^*

(F, F’) *w*/w*; +/UAS-sas.IR^GD1486^; bab1-Gal4, sas::SGFP/UAS-Dcr-2*

**S6 Fig**

(A, A’, A*, A*’) *w*/w*; UAS-RHG.miRNA/tub-Gal80^ts^; +/bab1-Gal4*

(B, B’, B*, B*’) *w^1118^/w*; UAS-p35/tub-Gal80^ts^; +/bab1-Gal4*

(C, C’, C*, C*’) *w*, UAS-Diap1/w*; +/tub-Gal80^ts^; +/bab1-Gal4*

(D, lane 1) *w*/w*; +/tub-Gal80^ts^; UAS-LUC.IR^TRiP.JF01355^/bab1-Gal4*

(D, lane 2) *w*/w*; UAS-RHG.miRNA/tub-Gal80^ts^; +/bab1-Gal4*

(D, lane 3) *w*, /w*; UAS-lacZ/tub-Gal80^ts^; +/bab1-Gal4*

(D, lane 4) *w^1118^/w*; UAS-p35/tub-Gal80^ts^; +/bab1-Gal4*

(D, lane 5) *w*, UAS-Diap1/w*; +/tub-Gal80^ts^; +/bab1-Gal4*

**S7 Fig**

*w^1118^/ w^1118^; +/+; +/+*

**S8 Fig**

(A, A’) *w*/w*; UAS-RHG.miRNA/+; +/bab1-Gal4*

(B, B’) *w^1118^/w*; UAS-p35/+^s^; +/bab1-Gal4*

(C, C’) *w*, UAS-Diap1/w*; +/*; +/bab1-Gal4*

(D, lane 1) *w*/w*; +/+; UAS-LUC/bab1-Gal4*

(D, lane 2) *w*/w*; UAS-RHG.miRNA/+; +/bab1-Gal4*

(D, lane 3) *w^1118^/w*; UAS-p35/+; +/bab1-Gal4*

(D, lane 4) *w*, UAS-Diap1/w*; +/+; +/bab1-Gal4*

**S9 Fig**

(B-B’’’, C-C’’’) *w^1118^/ w^1118^; +/puc-Stinger; puc^E69^-lacZ/+*

(D, D’) *w^1118^/ w^1118^; puc-Stinger/+; +/+*

(E, E’) *w^1118^/w*; puc-Stinger/UAS-eiger^W^; +/GMR-Gal4*

**S10 Fig**

*w^1118^/ w^1118^; puc-Stinger/+; +/+*

**S11 Fig**

(A, black line, closed dots) *w*, Ptp10D^1^/w^1118^; +/+; +/+*

(A, black line, open dots) *w*, Ptp10D^1^/w*, Ptp10D^EP1172^; +/+; +/+*

(A, dashed line, open dots) *w*, Ptp10D^1^/w*, Ptp10D^1^; +/+; +/+*

(B, black line, closed dots) *w*/w*; UAS-Dcr-2/tub-Gal80^ts^; UAS-LUC.IR^TRiP.JF01355^/bab1-Gal4*

(B, dashed line, open dots) *w*/w*; UAS-Dcr-2/tub-Gal80^ts^; UAS-Ptp10D.IR^GD115^/bab1-Gal4*

**S12 Fig**

(A, A’) *w^1118^/ w^1118^; +/+; +/+*

(B, B’) *w*, Ptp10D^1^/w*, Ptp10D^1^; +/+; +/+*

(C, C’) *w^1118^/ w^1118^; +/+; +/+*

(D, D’) *w*, Ptp10D^1^/w*, Ptp10D^1^; +/+; +/+*

*Fourth chromosomes of all flies are wild-type.
