# Supplementary material for: Sas-Ptp10D shapes germ-line stem cell niche by facilitating JNK-mediated apoptosis
Source: PLoS Genet. 2023 Mar 27;19(3):e1010684. doi: 10.1371/journal.pgen.1010684 (PMC10079222; doi:10.1371/journal.pgen.1010684)
Supplement: S2 File — (DOCX) [file pgen.1010684.s016.docx]

**S2 File. Sequence map of *P-puc-Stinger*.**

> P-puc-Stinger 15136 bp ds-DNA circular

tctggacgaagagcatcaggggctcgcgccagccgaactgttcgccaagcttgggctgcatcacgtaataagtgtgcgttgaatttattcgcaaaaacattgcatattttcggcaaagtaaaattttgttgcataccttatcaaaaaataagtgctgcatactttttagagaaaccaaataattttttattgcatacccgtttttaataaaatacattgcataccctcttttaataaaaaatattgcatactttgacgaaacaaattttcgttgcatacccaataaaagattattatattgcatacccgtttttaataaaatacattgcataccctcttttaataaaaaatattgcatacgttgacgaaacaaattttcgttgcatacccaataaaagattattatattgcataccttttcttgccataccatttagccgatcaattgtgctcggcaacagcatgctgcagcagatcTGATAAGGGTTGTGTCGTCGACGCGCCTCCTGCGCTGCCCGAGTGTTAATGTATTTTTGGCCACCACTGTGCCCTGGCTCGATGATGACTCATCGCCATTACGTTTCGTTGTTCGTTCACAATGCAATGTGGAACACAGAGAGCGAGATGGCGAGAGAGTGGGTGGGCGATAGGTGGTGGTGGGTGGGTGGATGGATGGATGGTTGGGTGAACGGTACAGCCGCATCGATTTTTCTGTTGGCTTCTTATTGTTGCTATTTATTGCGCCGCCAACGGCACTTCTCACCCCTCTCTTTTCCTCCGGCGCTGTTTCGTTTTGGCATATGTTCTATTTTTGTAAACATTTCGGTTCCTGTTCATGACTCATCGATTTGGTTTTTTCTTCTTTTAGTGGGTTATCCTTGTGCGTGCGGCCTCGGCTGTTGGTGTTTTATGGTCGTTGTTGTTGTTGTGGATTTCAGGTGCGGGGCGCAGCAACAGCTGGTTAATATCGCTTCCTCTCTCGCTTGCCTGCGCTCTTTATTTTATTTGCATCTGTCGGATCAGAATCCTTGGTTCAGACTTCTTTTGACCGCTACACAGAAAGAAATCTAAGTAACAAATTTTTAAAATGGGATTAAAGCTTTAAAAAAATTTTAAAGTGTGCTTCTATTAATTGTTGCTCCTTAACTTCGCCGCTTTTATACATGCTTAAGTATTATTTTTATAAATGCTCTACCTCTCTCGCTCACTCACACCCGCGCAGCAACACATTAGTACTCAGGCACGAAGTAACGGTGGGTGGGGAGACGGACAAGAGAAAGAAGAAGCAAAAGGAGGTGGCGGCGGCGGCGGGAAAGAAGAAGCAGGGAAAAGTATACGTACAATTTTGTACTGAGTGCATGACTCATGATTTCGCTTCTCTGTTTGTGTTTGTGAGTTTGTGTGTATTTGCCCCGGCCTTTTGCGCATTCGTTTTTGGTTTGCCGTCGTCACCTTATGCCTATTATTATTGTTTTATTTCGCCTGAATATATGCTGATCCCCTTCTTAGTGAGCTTCCTCTTCTACCGTACAACTTATGATTTCTCATTGTTCCGCTGCTTTTCGTTTTCCATTCATTATAACAGCACTTTGTGTGCGCGGGGGTGGCGGGGTGTTGGGGGATGTGGGGAAAGAGGAAGCACCGCTATTTTGAAGCAGAGCTTAAAATGAACTCAACGCCGGCAGAGGCAGCGGCAGTGGCAGCGGCACCGGCAGAGGCTCATTAGGCGACGGTAGAGTTCGAGGCCCCAATTGTTGTTGTAGTTGTAGTTGTCGGATAATGCTAATGTCATTATGCTGTGTCATTAACATGAACGATAAACCAAACCGAGCCACTTGGCCTAGAAAGCAATCAAAGCCTCAATTGTCAGGAAATTGAGCGGTATTTCAATCGGTAGTATTCGCTCTATTTTACTTGTAATGGAGATTTAAGCGTTACAAGGATTTTTCAGATATCCCAAAAAGTTAAGAACTAAAAAATTAAAGACCAATTTACTAATAACAAATGAAATTTGCATCTATTTCTGTACATATGTATGCAATGTTTTGTTTCATAAACCAAGAAAAAGAGGTAAAATCTTACTTCCTTCAAGGTATCTGACATTTTTAAACACAAGGCCATAATTATAAACAAACGTTCCGCTTAAATTGGCCAAGAATACCAGATGCTGAGGTAAATAAACATAATAGCAAGGTTTAAATTCGAAATAGAGATCTCTCACAGCAATGCTCGATCTATTATCTATTATCTATTATCTAGGGGCTTTCTAGCCGATTCCACATCCTTTCCTACGATTGCCATCCAATTTGTCAACTTATCGCCGCGATGTCTGTACTGACTGTACCCATATTATTTGCGCGCCAAGCTATCAAGTTATCAAGCCGTCAACTTTTATCTGCCAACGACGGAGAGGCCTCTTTGGCGAACTAACTTAATCTACAACGGAGCATACAAACACACATATGTACATATATAGATTTGTATATATACGGTTACAGGTTACGTTTACGGGGCATTCGAAGTACAATTACGGGTTGTGGACACACGGTCGTAGAAGCAGCTAAGCAAACTGAAAGCTGAGGCCCCTCGAGAACATTTGGCGATAGTCACATTCTATATACATACATATATACAATTGGACAGCTGATTAGATAAAACTATGAATGCATATGTTCAAATATATACCGAGAGTTTAGAAAATAGATTGTTCTAATATAAAAAGTCAATATCGTTGTCCCAAGTAACTCAGCCAAAAAGTACTCAGTATAAAATGAGAGAACCCAAAAAAAATCAAAATCCTTCGGCTAATGAGTAATCGAGAATCCGTTGGCAGACAACAAACAAAAAAGAATTAACCGAAACAACAACCAAAAACCCGAGGTGACATTTTTGTTGGCCCCCGTGCGCATAATTTCAATATTCGTCAGCCGGCTATTTTTAAAATGCAAAAATCGACCAAGCGAAAAAAAATGAAAATATTCGCACAGGAATTTCAAATTTGAAATTTGCGTTGCGGCTTCGTTCTTTTTATTGTTGTATCGAATGTATAAGGCTCTTAATCATTTTAAAAGGGAACCCGAGGCCCACACTTCCTCGTTTTTCTCTACAGAGAAAAAAATATGATTAATATACACATGAATTATTTAGAATATCACTTTTAAGATCGTAAGATGGGATCGTTATTACTTCAACGAAAAAGTCTCGTCAATGGGAGGGTATACAAAAACAACTCATTGTGTACTTATTCATCAATACCTTCCTTTTGAGACTTTTGCGCCCGCGCACTCTGTATTTTCCAAGTGTTTGATTTGCGCGCAATTTGTTTATGAAACATTCCGTTTGTTGTTATTGCCGCTGCGCATGCTCTTCGTGAATCACCCGAGGAGAGCCCTGATCCATCTACCTGCCTCCCTCACTCACTCACTCACTACCCACTATTCACCCCGACCCGACCCGCTTAAGTTGAAGGCGACTTCAAAGTGCAGGCTGTCTTGTTAGGGGGGCTTTGTTGTTTCCGTATGACTCGACACGTCAAAGTCGAGATTCGCCAAGTTGAAGGTCAAGTTGAGATCCCACTCTCTTTGTTTTCGATGATTCAGCGGGGTCTTATCGCCATTTAAGTGTCCTGGCACTGGCACCTGCCGCACTCAGACTCACACTGCGGCTGTCTCTTTCGGTCGGCATACGGCCGTCTCTTTCTGGCTGAATCAGAAATCCGTATGAGTAAGCGGAGTATTCTTAATGATAAACGGTCGCCGAAAGTAATTAAATATTTCGAACATAAACAAAACTACTTGTTGCTTGACAACGTGCTTTGGTTAAAGCAGCCAGCAGTATAAAAATAACATCAAGCTAAAGAAGTTCATAACAAACTGAGTGTTGAAAAATGGTTACTTTATTAGGTAATTAGAAGTGTTAAATGCCATTCAAGTAAAATTTACTATATTTTTAAAAATATTCTTAGTTTAATTGTTTTCTTATGATCCTTAAGGACATTTCCTTGTGTTATTACCAACTATGCATCCTTGGCTCTTGTGCTATTTCCCTGAACATTGCTGTAAAACATAAAACAGCTCATTCATATATCATGTTCCTCCTGCTCAAAGGTTGTTTCTGTTTTCGATTCTGATTCCGATTCTGTTGCCGTTTCTGTTTCTGGCCATGACTCAGAGGGAGTTGCGAGAGTTTCGCGCCCTCGAACACATTTGGTTGACCCTTGGGTCCGAGATCCGGGACTTAATCATCACGAGAAAAAGTGAAATGATGCATTGGGCAAGCGGACATTGAGAACAAGAACCGAAAACGACGCGACAAGTCGTCATCGTCGTTGGCTGGCGAGACGGCGATACCATCCACCATATAACCCCATTTGATCCCATACCATCGCCCTGGCATCCCAGCTGTCAGACAGTCGTCTGCCACTTGGCCCACTTCTGAGCCGAAATCATCAGATGACGCACCCGGGGGCCGGACAGTAAAATGAAGAAAAAAAAGAGACATCGCTACTGTTGCACTTAATCATACATTCATTGGTGTAAGAATTCGGTGACTACGACTACCCAAAGTTGGCATTACGCATCGCCCCCGAAATGGAGTCACGACTCTCAGCCCTTTGGAATGGTGGCAACGTGTACTGCCCCATTATAGGCACGACACGACGGGATCCATCAGTCATTCACAGTCATCGGCGGAATATGTGCTCCGGTGAACTAAGTTGTTTAGTCATACGCATGCAAGGCTCTATTGGCGATTAGTAATTCCCCACAGCATATATGGCCGCATATTTGTCACGAACAATAAACAATTACCAAAGCCCGGCTTCCCAATAATCGATCAAAGCGTCCGCTTTGAGTTCCGTTCTCTGGGATCGAAACTTAAACAGAAAAATGCAAAAATTACGGAAAAAGATAAACAAACAGATTATTTGTTGGCTTTCTTTACGCACATATACCCATGATGACTAGTTTAAAGAAAACAGTTATATGATGCTATAAATGATACAGCATTTTACAAAATTGTAAACTAGTTTTTCCTAAAAATGAACTTTATTTGATTTCTCAACACTTCTAGTTCTCAGTTCTTAAATTGAACCCCAGTCCAATTAGCCCGAACAACTTCATAGTGACTCAAACTAGACTTTTGCGAAATTCATTCATCATTAACTAGTGCTAGTTGTGGCTAGTTTTTGCGAAACTCCTTAGAATTAGTCCCCCTGTCATTATGCATTATAATATCTTGGACCGTTCTAGCCCACATATGATATGAGTCATGTGGCAGGCGCGTTTACGGCGCGTTTTATGCGCGATTTTAACTGTCTGAGCGACGGGGTCATTAACTAAGAGGaattcgccggcgcgccggtaccccgcggccgctagcggatccctcgaggagcgccggagtataaatagaggcgcttcgtctacggagcgacaattcaattcaaacaagcaaagtgaacacgtcgctaagcgaaagctaagcaaataaacaagcgcagctgaacaagctaaacaatctgcagccaagctgatcctctagggtacgcgctagagtcgagaggcctgtttaaacgatccaccggtcgccaccatggtgagcaagggcgaggagctgttcaccggggtggtgcccatcctggtcgagctggacggcgacgtaaacggccacaagttcagcgtgtccggcgagggcgagggcgatgccacctacggcaagctgaccctgaagttcatctgcaccaccggcaagctgcccgtgccctggcccaccctcgtgaccaccctgacctacggcgtgcagtgcttcagccgctaccccgaccacatgaagcagcacgacttcttcaagtccgccatgcccgaaggctacgtccaggagcgcaccatcttcttcaaggacgacggcaactacaagacccgcgccgaggtgaagttcgagggcgacaccctggtgaaccgcatcgagctgaagggcatcgacttcaaggaggacggcaacatcctggggcacaagctggagtacaactacaacagccacaacgtctatatcatggccgacaagcagaagaacggcatcaaggtgaacttcaagatccgccacaacatcgaggacggcagcgtgcagctcgccgaccactaccagcagaacacccccatcggcgacggccccgtgctgctgcccgacaaccactacctgagcacccagtccgccctgagcaaagaccccaacgagaagcgcgatcacatggtcctgctggagttcgtgaccgccgccgggatcactctcggcatggacgagctgtacaagagcaggcacagaaggcatcgccagcgctctaggagccgcaatcgcagccgaagtcgcagcagtgaacgaaaacgccgtcaacggagccgaagtcgcagcagtgaacgaagacgctaacttgtacaagtaaagcggccgcgactctagatcataatcagccataccacatttgtagaggttttacttgctttaaaaaacctcccacacctccccctgaacctgaaacataaaatgaatgcaattgttgttgttaacttgtttattgcagcttataatggttacaaataaagcaatagcatcacaaatttcacaaataaagcatttttttcactgcattctagttgtggtttgtccaaactcatcaatgtatcttaactagtcacgtaataagtgtgcgttgaatttattcgcaaaaacattgcatattttcggcaaagtaaaattttgttgcataccttatcaaaaaataagtgctgcatactttttagagaaaccaaataattttttattgcatacccgtttttaataaaatacattgcataccctcttttaataaaaaatattgcatactttgacgaaacaaattttcgttgcatacccaataaaagattattatattgcatacccgtttttaataaaatacattgcataccctcttttaataaaaaatattgcatacgttgacgaaacaaattttcgttgcatacccaataaaagattattatattgcataccttttcttgccataccatttagccgatcaattctagtatgtatgtaagttaataaaacccttttttggagaatgtagatttaaaaaaacatattttttttttattttttactgcactggacatcattgaacttatctgatcagttttaaatttacttcgatccaagggtatttgaagtaccaggttctttcgattacctctcactcaaaatgacattccactcaaagtcagcgctgtttgcctccttctctgtccacagaaatatcgccgtctctttcgccgctgcgtccgctatctctttcgccaccgtttgtagcgttacctagcgtcaatgtccgccttcagttgcactttgtcagcggtttcgtgacgaagctccaagcggtttacgccatcaattaaacacaaagtgctgtgccaaaactcctctcgcttcttatttttgtttgttttttgagtgattggggtggtgattggttttgggtgggtaagcaggggaaagtgtgaaaaatcccggcaatgggccaagaggatcaggagctattaattcgcggaggcagcaaacacccatctgccgagcatctgaacaatgtgagtagtacatgtgcatacatcttaagttcacttgatctataggaactgcgattgcaacatcaaattgtctgcggcgtgagaactgcgacccacaaaaatcccaaaccgcaatcgcacaaacaaatagtgacacgaaacagattattctggtagctgtgctcgctatataagacaatttttaagatcatatcatgatcaagacatctaaaggcattcattttcgactacattcttttttacaaaaaatataacaaccagatattttaagctgatcctagatgcacaaaaaataaataaaagtataaacctacttcgtaggatacttcgttttgttcggggttagatgagcataacgcttgtagttgatatttgagatcccctatcattgcagggtgacagcggacgcttcgcagagctgcattaaccagggcttcgggcaggccaaaaactacggcacgctcctgccacccagtccgccggaggactccggttcagggagcggccaactagccgagaacctcacctatgcctggcacaatatggacatctttggggcggtcaatcagccgggctccggatggcggcagctggtcaaccggacacgcggactattctgcaacgagcgacacataccggcgcccaggaaacatttgctcaagaacggtgagtttctattcgcagtcggctgatctgtgtgaaatcttaataaagggtccaattaccaatttgaaactcagtttgcggcgtggcctatccgggcgaacttttggccgtgatgggcagttccggtgccggaaagacgaccctgctgaatgcccttgcctttcgatcgccgcagggcatccaagtatcgccatccgggatgcgactgctcaatggccaacctgtggacgccaaggagatgcaggccaggtgcgcctatgtccagcaggatgacctctttatcggctccctaacggccagggaacacctgattttccaggccatggtgcggatgccacgacatctgacctatcggcagcgagtggcccgcgtggatcaggtgatccaggagctttcgctcagcaaatgtcagcacacgatcatcggtgtgcccggcagggtgaaaggtctgtccggcggagaaaggaagcgtctggcattcgcctccgaggcactaaccgatccgccgcttctgatctgcgatgagcccacctccggactggactcatttaccgcccacagcgtcgtccaggtgctgaagaagctgtcgcagaagggcaagaccgtcatcctgaccattcatcagccgtcttccgagctgtttgagctctttgacaagatccttctgatggccgagggcagggtagctttcttgggcactcccagcgaagccgtcgacttcttttcctagtgagttcgatgtgtttattaagggtatctagcattacattacatctcaactcctatccagcgtgggtgcccagtgtcctaccaactacaatccggcggacttttacgtacaggtgttggccgttgtgcccggacgggagatcgagtcccgtgatcggatcgccaagatatgcgacaattttgctattagcaaagtagcccgggatatggagcagttgttggccaccaaaaatttggagaagccactggagcagccggagaatgggtacacctacaaggccacctggttcatgcagttccgggcggtcctgtggcgatcctggctgtcggtgctcaaggaaccactcctcgtaaaagtgcgacttattcagacaacggtgagtggttccagtggaaacaaatgatataacgcttacaattcttggaaacaaattcgctagattttagttagaattgcctgattccacacccttcttagtttttttcaatgagatgtatagtttatagttttgcagaaaataaataaatttcatttaactcgcgaacatgttgaagatatgaatattaatgagatgcgagtaacattttaatttgcagatggttgccatcttgattggcctcatctttttgggccaacaactcacgcaagtgggcgtgatgaatatcaacggagccatcttcctcttcctgaccaacatgacctttcaaaacgtctttgccacgataaatgtaagtcttgtttagaatacatttgcatattaataatttactaactttctaatgaatcgattcgatttaggtgttcacctcagagctgccagtttttatgagggaggcccgaagtcgactttatcgctgtgacacatactttctgggcaaaacgattgccgaattaccgctttttctcacagtgccactggtcttcacggcgattgcctatccgatgatcggactgcgggccggagtgctgcacttcttcaactgcctggcgctggtcactctggtggccaatgtgtcaacgtccttcggatatctaatatcctgcgccagctcctcgacctcgatggcgctgtctgtgggtccgccggttatcataccattcctgctctttggcggcttcttcttgaactcgggctcggtgccagtatacctcaaatggttgtcgtacctctcatggttccgttacgccaacgagggtctgctgattaaccaatgggcggacgtggagccgggcgaaattagctgcacatcgtcgaacaccacgtgccccagttcgggcaaggtcatcctggagacgcttaacttctccgccgccgatctgccgctggactacgtgggtctggccattctcatcgtgagcttccgggtgctcgcatatctggctctaagacttcgggcccgacgcaaggagtagccgacatatatccgaaataactgcttgtttttttttttaccattattaccatcgtgtttactgtttattgccccctcaaaaagctaatgtaattatatttgtgccaataaaaacaagatatgacctatagaatacaagtatttccccttcgaacatccccacaagtagactttggatttgtcttctaaccaaaagacttacacacctgcataccttacatcaaaaactcgtttatcgctacataaaacaccgggatatattttttatatacatacttttcaaatcgcgcgccctcttcataattcacctccaccacaccacgtttcgtagttgctctttcgctgtctcccacccgctctccgcaacacattcaccttttgttcgacgaccttggagcgactgtcgttagttccgcgcgattcggttcgctcaaatggttccgagtggttcatttcgtctcaatagaaattagtaataaatatttgtatgtacaatttatttgctccaatatatttgtatatatttccctcacagctatatttattctaatttaatattatgactttttaaggtaattttttgtgacctgttcggagtgattagcgttacaatttgaactgaaagtgacatccagtgtttgttccttgtgtagatgcatctcaaaaaaatggtgggcataatagtgttgtttatatatatcaaaaataacaactataataataagaatacatttaatttagaaaatgcttggatttcactggaactagaattaattcggctgctgctctaaacgacgcatttcgtactccaaagtacgaattttttccctcaagctcttattttcattaaacaatgaacaggacctaacgcacagtcacgttattgtttacataaatgattttttttactattcaaacttactctgtttgtgtactcccactggtatagccttcttttatcttttctggttcaggctctatcactttactaggtacggcatctgcgttgagtcgcctccttttaaatgtctgaccttttgcaggtgcagccttccactgcgaatcattaaagtgggtatcacaaatttgggagttttcaccaaggctgcacccaaggctctgctcccacaattttctcttaatagcacacttcggcacgtgaattaattttactccagtcacagctttgcagcaaaatttgcaatatttcatttttttttattccacgtaagggttaatgttttcaaaaaaaaattcgtccgcacacaacctttcctctcaacaagcaaacgtgcactgaatttaagtgtatacttcggtaagcttcggctatcgacgggaccaccttat

misc_feature 62..457

/locus_tag="gypsy"

/label="gypsy"

misc_feature 498..5323

/locus_tag="puc intron"

/label="puc intron"

misc_feature 5392..5524

/locus_tag="hsp70 promoter"

/label="hsp70 promoter"

misc_feature 5593..6426

/locus_tag="eGFP-NLS CDS"

/label="eGFP-NLS CDS"

misc_feature 6689..7075

/locus_tag="gypsy"

/label="gypsy"
