# Supplementary material for: Sas-Ptp10D shapes germ-line stem cell niche by facilitating JNK-mediated apoptosis
Source: PLoS Genet. 2023 Mar 27;19(3):e1010684. doi: 10.1371/journal.pgen.1010684 (PMC10079222; doi:10.1371/journal.pgen.1010684)
Supplement: Attachment — Submitted filename: Response to reviewers.pdf [file pgen.1010684.s018.pdf]

We would like to thank the reviewers for helpful comments, which have led us to improve our manuscript substantially. Modifications are indicated as track changes in the revised manuscript.

Responses to Reviewers' comments:

Reviewer #1:

In this study, the authors investigate the role of a signaling cascade mediated by the transmembrane protein, Sas, and its receptor, Ptp10D, in the development of the Drosophila ovarian germline stem cell niche. The study begins with the observation that the adult ovaries of Ptp10D[1] mutant homozygotes have more germline stem cells than wildtype. Using standard genetic tools in the field to restrict RNAi expression in time and space, they demonstrate that Ptp10D and sas are required in the surrounding somatic cells specifically during a midlarval stage. Subsequent analysis provides evidence that the effect on adult stem cell number is caused by a role for this pathway in determining niche shape by promoting JNK-mediated apoptosis of apical cells during development via inhibition of egfr. The study is well-designed and, in general, the data provide clear support for the model. However, several issues would be important to address prior to

1. The RNAi phenotypes in Fig. 1E-F are subtle, especially compared to the phenotype in the Ptpn10D null (Fig. 1D) and it is not clear if this is because the RNAi knockdown is incomplete or another reason. To test the efficiency of knockdown, the authors should use the anti-Ptp10D antibody and Sas::SGFP line in combination with the RNAi.

<u>Response:</u>
We thank the reviewer for careful assessment and important comment. We agree that the phenotypes of *Ptp10D*-RNAi and *sas*-RNAi flies are weaker than those of *Ptp10D*^{1} homozygous mutants. Following the reviewer's suggestion, we checked the efficiency of *Ptp10D*-RNAi and *sas*-RNAi in gonadal apical cells using anti-Ptp10D and anti-GFP (for Sas::SGFP) antibodies. As pointed out by the reviewer, both RNAis significantly suppressed expression of their targets but they were not complete. We have now added these data in the revised manuscript as S7 Fig as follows:

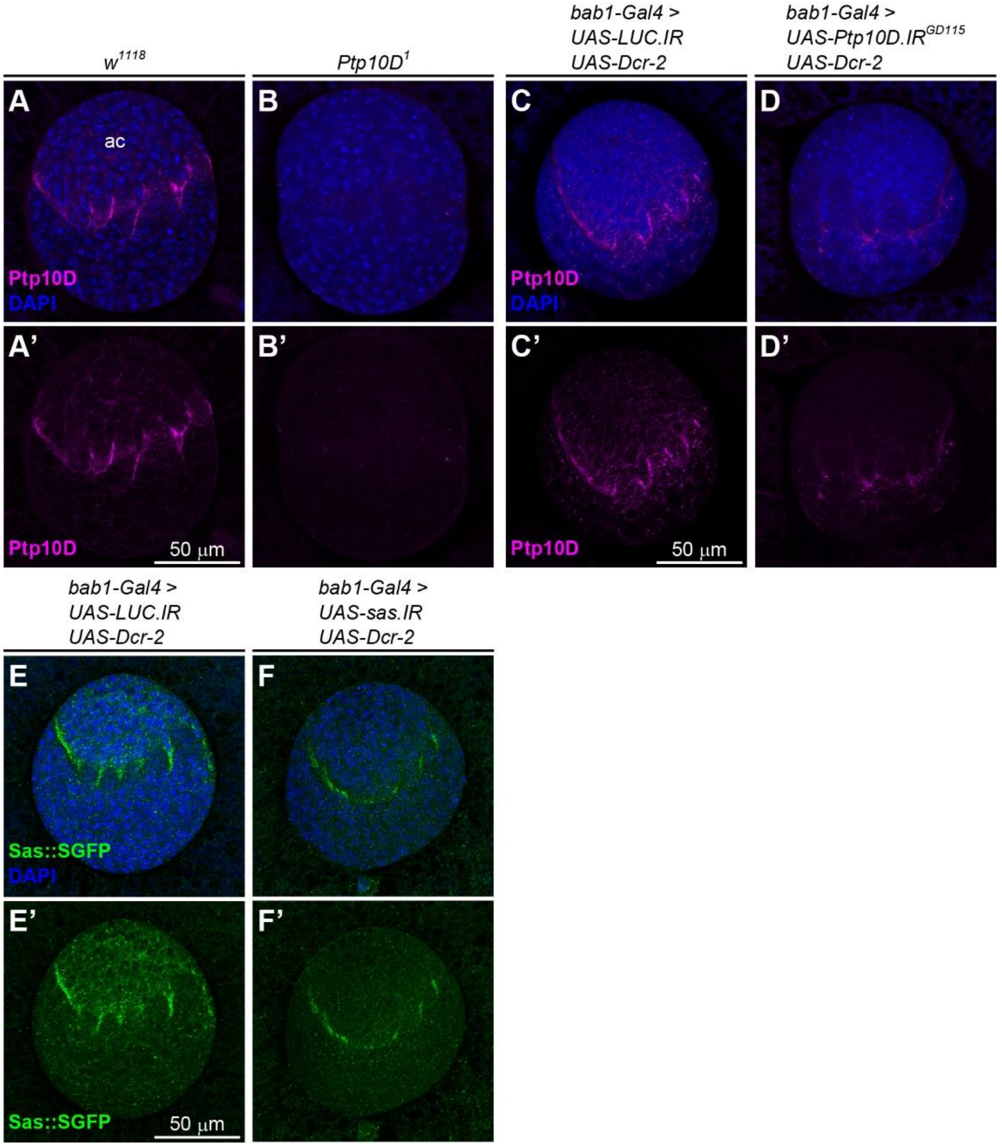

S7 Fig. Validation of antibodies and RNAi lines.

(A-D) Female gonads of indicated genotypes (upper) at wondering L3 stages are labeled with DAPI (blue) and anti-Ptp10D antibody (magenta) (A-D), and anti-GFP antibody for Sas::SGFP (green) (E and F) are shown. The cell layer located at the opposite side of the fat body adherent surface are shown. Images are processed by the Z-stack projection of two sections (corresponding to 3 µm thickness). (A'-D') Magenta channels of (A-D). (E' and F') Green channels of (E and F). Scale bars, 50 µm. *Ptp10D^{1}* exhibited a complete loss of Ptp10D staining in gonad (A and B). Knockdown of *Ptp10D* by *bab1-Gal4* reduced the Ptp10D staining especially in apical cells (C and D). Knockdown of *sas* by *bab1-Gal4* reduced Sas::SGFP (E and F) in apical cells.

(Results, line 185-187)

We also confirmed that *Ptp10D^{1}* abolished Ptp10D protein expression in gonads and that

bab1-Gal4-mediated knockdown of *Ptp10D* or *sas* reduces Ptp10D protein expression or Sas::SGFP in apical cells, respectively (S7 Fig).

2. Likewise, the effect of Ptp10D RNAi knockdown on the rate of egg production is modest. Expression of Sas RNAi with bab1 had a stronger effect on GSC number—one that looks more similar to the effect in Ptp10D[1] mutants. What is the rate of egg laying in these mutants? Quantifying egg laying in additional mutants would help strengthen the correlation between increased GSC number and decreased egg laying.

<u>Response:</u>

We agree with the reviewer that it is important to examine the correlation between increased GSCs and decreased egg laying using additional mutants. However, unfortunately, most females of *sas*-RNAi with *bab1-Gal4* exhibited severe abnormalities in their leg morphology and behaviors, which would affect nutritional status and mating behaviors. Therefore, we took alternative approach; we quantified the egg laying of trans-heterogenous mutants of *Ptp10D*^{1} and *Ptp10D*^{EP1172}, which exhibited moderate increase in the number of GSCs (Fig 1D) and intermediate phenotypes in egg laying. These data further support the correlation between increased GSC number and decreased egg laying. We have now added these *Ptp10D*^{1}/*Ptp10D*^{EP1172} data as Fig.6A and A' as follows:

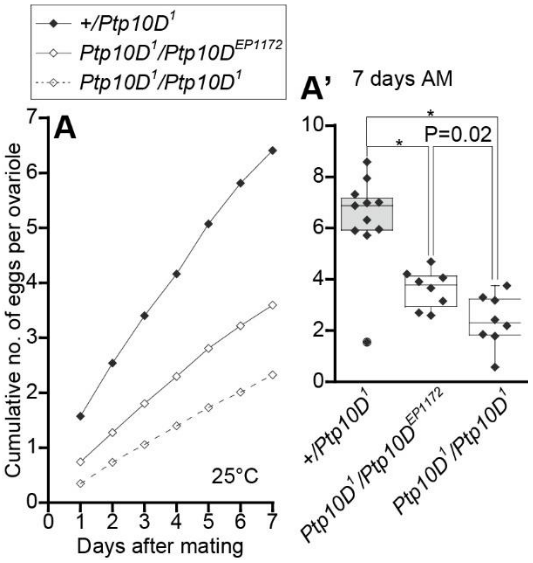

Fig 6A and A'

(A and B) Line graphs represent means of cumulative numbers of eggs per ovarioles laid by single females after mating with single wild-type *Canton-S* males. (A) Data values from control

+/Ptp10D^{1} (black line and closed dots in A), *Ptp10D*^{1}*/Ptp10D*^{EP1172} (black line and open dots in A), and *Ptp10D*^{1}*/Ptp10D*^{1} (dashed line and open dots in A) at 25 degrees.

(Results, line 272-275)

We also found that trans-heterozygous females for *Ptp10D*^{1} and *Ptp10D*^{EP1172}, which moderately increased the number of GSCs (Fig 1D), exhibited intermediate phenotype in egg production (Fig 6A), suggesting that increased GSCs number correlates with decreased egg laying.

3. As a minor comment (optional to address), displaying the rate of egg laying per ovariole rather than per female has advantages but is also unconventional. Can a separate graph or table be provided that shows the number of eggs laid per female so that these data can be more directly compared to egg laying assays in other papers?

Response:

We thank the reviewer for the comment. As pointed out by the reviewer, "egg laying per ovariole" used in our experiment has an advantage for indicating the egg production of germarium but not a standard way. Following the reviewer's suggestion, we have now added "egg laying per female" data in the revised manuscript as S13 Fig as follows:

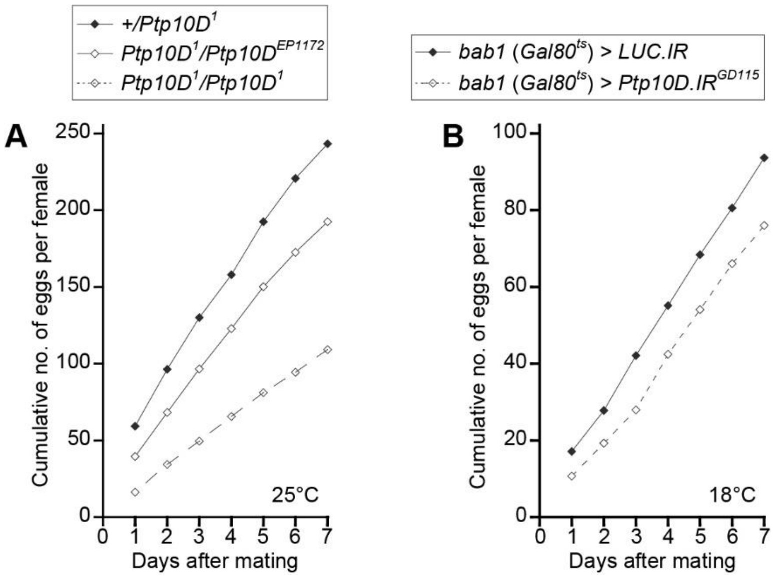

S13 Fig

(A and B) Line graphs represent means of cumulative numbers of eggs laid by single females after mating with single wild-type *Canton-S* males. (A) Data values from control *+/Ptp10D*^{1} (black line and closed dots in A), *Ptp10D*^{1}*/Ptp10D*^{EP1172} (black line and open dots in A) and

Ptp10D¹/Ptp10D¹ (dashed line and open dots in A) at 25 degrees. (B) Data values from control *UAS-LUC.IR* (black line and closed dots in B) and *UAS-Ptp10D.IR* (dashed line and open dots in B) by *bab1-Gal4*/TARGET at 18 degrees. (A' and B').

(Results, line 269-270)

To address this, we measured the number of egg laying (S13 Fig) and ovarioles per female and calculated the number of eggs per ovariole as a reproductive capacity (Fig 6).

4. The effect on apoptosis suggests that there should be more cap cells in Sas and Ptp10D mutants. Is this the case?

Response:

We thank the reviewer for the important comment. Indeed, we found that the number of cap cells was slightly (approximately 1 cell) increased in *Ptp10D¹* mutant. However, it is considered that this increase in the number of cap cells is not directly due to apoptosis of apical cells because the progenitor cells of cap cells are not apical cells but interstitial cells (Fig. 1A). We have now added this data in the revised manuscript as S6G Fig as follows:

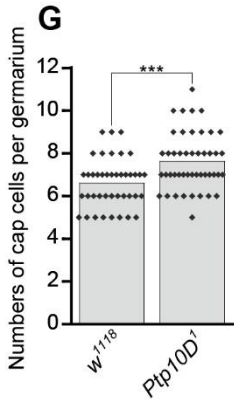

S6 Fig

(G) Bar graph overlaid with beeswarm plots represent numbers of cap cells per germarium. Genotypes are indicated at the bottom. P-values (***P < 0.0001) for Mann-Whitney U test is shown.

(Results, line 178-181)

We only found that the number of cap cells was slightly (approximately 1 cell) increased

in *Ptp10D[1]* (S6G Fig). However, it is likely that one-cell increase in the number of cap cells does not have significant impact on expanding the surface of cap cell-cluster.

5. An important test of the proposed model would be to determine whether the miniCic::mCherry signal is altered in the Ptp10D and/or Sas mutants.

<u>Response:</u>

We agree with the reviewer that it is important to confirm that ERK activity is altered (elevated) in the *Ptp10D* mutants. To test this, we quantified the ERK activity using the anti-phosphorylated ERK antibody (pERK) staining. Indeed, we observed elevated pERK staining in the apical cells of *Ptp10D[1]* mutants (please see Fig 3A-C, below). We have now added these data in the revised manuscript as Fig 3A-C as follows:

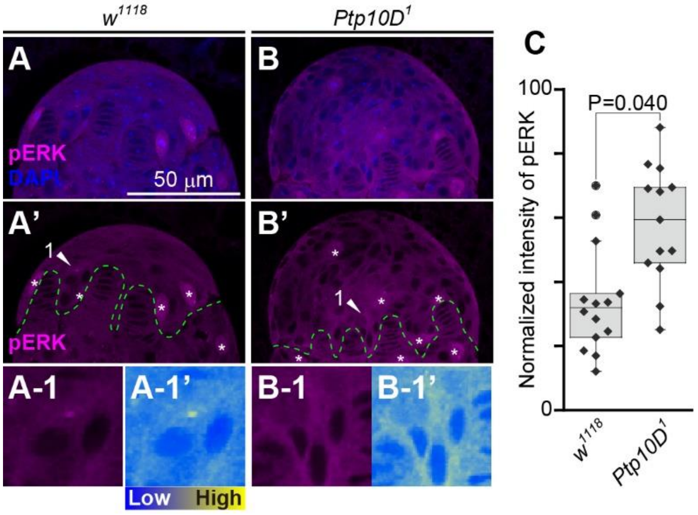

Fig 3A-C

(A and B) Female gonads of late wandering larvae in w^{1118} and *Ptp10D[1]* homozygotes were labelled with anti-pERK antibody (magenta) and DAPI (blue). (A’ and B’) Magenta channels of (A and B). Asterisks in (A’ and B’) indicate non-specific staining of anti-pERK in mitotic cells. Green dashed lines in (A’ and B’) indicate the boundaries between apical cells and terminal filament cells. Apical cells indicated by arrowheads 1 in (A’ and B) are shown in (A-1 and B-1). Heatmap images of (A-1 and B-1) are shown in (A-1’ and B-1’). (C) Boxplot overlaid with beeswarm plot represents the normalized intensity of pERK staining. Data values from w^{1118} and *Ptp10D[1]* are plotted. P-value for Mann-Whitney U test are shown.

(Results, line 194-197)

Indeed, *Ptp10D*^{1} exhibited elevated Egfr signaling in gonadal apical cells, as visualized by anti-phospho-ERK (pERK) staining (Fig 3A-C), and stage-specific knockdown of *Egfr* in gonadal apical cells by *bab1-Gal4* significantly suppressed the abnormal shape of the cap cell-cluster in *Ptp10D*^{1} (Fig 3D).

6. I might be missing something, but I have not seen the raw data for this study. The review invitation included a link to view the manuscript, but that just prompted a download of a PDF file that contained the manuscript and figures; no raw data was included in that PDF.

Response:

We have added raw dataset as S17 Fig.

Reviewer #2:

In Drosophila oogenesis, a cluster of cap cells form the niche for 2-3 germline stem cells (GSCs). In analyzing a viable mutant of Ptp10D, the authors discovered additional GSCs, an abnormal cap cell cluster structure and reduced fecundity. The phenotype was suppressed by heterozygosity for EGFR, suggesting that Ptp10D controls cap morphology via a similar pathway to cell competition where EGFR inhibits JNK-mediated apoptosis.

Using Gal4 drivers, the authors attempt to determine the cell specific requirement for Ptp10D and its ligand Sas. While they are able to show that the genes are required somatically, determining the exact cell type is a bit complicated due to the complex patterns of the Gal4 drivers. They conclude that the genes are required in the apical cells, since tj-GAL4 does not recapitulate the phenotype with UAS-Ptp10D RNAi and tj is not expressed in the apical cells, whereas the bab-1 and c587 drivers are. The bab-1-Gal4 shows the strongest effect, and the strongest effect was observed by expressing RNAi one day before the wandering larval stage.

They find that EGFR signaling via a Cic reporter is enriched in the distal apical cells adjacent to the terminal filaments. However, this does not seem to make sense in light of their proposed model. In their model (Figure 7), active Ptp10D should reduce EGFR signaling. This needs to be explained further.

They go on to examine apoptosis, and find a modest effect on cap cell structure using RHGmir and p35. They examine JNK signaling via puc overexpression and signaling and see an effect, suggesting that JNK regulates apoptosis to affect cap morphology.

This is an interesting manuscript pointing to a role for using the cell competition mechanism in tissue morphology. While the effects on oogenesis are clear, there are still a number of missing links that need further clarification. These are itemized below.

Major issues:

1. The cellular basis for the abnormal shape of the cap cell cluster is not described. Are there more cells? Are the cells a different shape? Or is anchorage to other cells disrupted? Is there a problem with the sheath? Some insight along these lines would be very helpful.

Response:

We thank the reviewer for summarizing our study and pointing out an important issue. We agree that it is important to analyze the cellular basis of abnormal cap cells in *Ptp10D* mutants. Following the reviewer's suggestion, we quantified the number of cap cells and

analyzed the morphology of cap cells and surrounding muscle sheath. We used anti-Lamin C antibody and anti-En antibody as differentiation markers, anti-*D*E-Cadherin antibody as an adhesion marker, and phalloidin as an actin marker. We found that the number of cap cells was slightly increased in *Ptp10D*^{1} mutants. However, it is considered that this increase in the number of cap cells does not have strong impact on the abnormal shape of the cap cell cluster, as one-cell increase of cap cells may not sufficiently expand the surface area of cap cell-cluster. In addition, we did not find any significant alterations in the morphology of cap cells and the surrounding muscle sheath. We have now added these data as S6 Fig in the revised manuscript as follows:

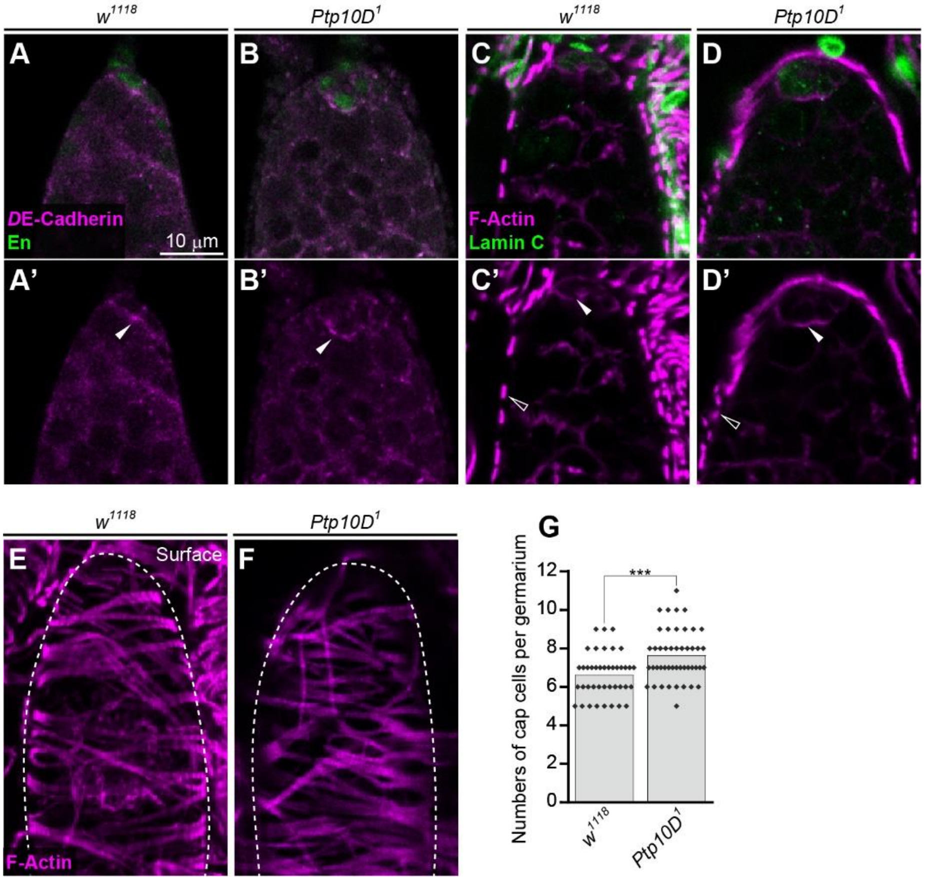

S6 Fig

(A-E) Proximal regions of female germaria 1 day after eclosion without mating are shown. Germaria are labeled with anti-DE-Cadherin (magenta) and anti-En antibody (green) (A and B), phalloidin (magenta) and anti-Lamin C (green) (C and D), and phalloidin (magenta) (E and F). Proximal is to the top. Germaria from *w*^{1118} (A, C, and E) and *Ptp10D*^{1} (C, D, and F) are shown. (A'-D') Magenta channels of (A-D). (E and F) Confocal sections of basal surfaces corresponding to surrounding muscle sheath are shown. White dashed lines in (E and F) indicate outlines of germaria. Scale bar in (B) is 10 μm, and applicable for (B-F). *Ptp10D*^{1} did not exhibit alterations of cap cells in differentiation (En and Lamin C in B and D). cell adhesion

(*D*E-Cadherin in B), and actin cytoskeleton (D). The arrangement of muscle sheath was not disrupted in *Ptp10D*^{1} (F). (G) Bar graph overlaid with beeswarm plots represent numbers of cap cells per germarium. Genotypes are indicated at the bottom. P-values (***P < 0.0001) for Mann-Whitney U test is shown.

(Results, line 174-181)

We further examined whether morphological changes of cap cells occurred in *Ptp10D*^{1}. However, we did not found alterations in differentiation (S6A-D Fig), cell adhesion (S6A and B Fig), and actin-cytoskeleton (F6C and D Fig). We also found that *Ptp10D*^{1} flies did not show defects in the structure of muscle sheath (S6E and F Fig), the primordial cells of which are gonadal apical cells (Fig 1A) (9). We only found that the number of cap cells was slightly (approximately 1 cell) increased in *Ptp10D*^{1} (S6G Fig). However, it is likely that one-cell increase in the number of cap cells does not have significant impact on expanding the surface of cap cell-cluster.

2. What is the relationship between the apical cells and the future cap cells? How do the authors propose that the increase/survival of apical cells leads to the abnormal cap cell cluster?

Response:

We thank the reviewer for the important comment. Indeed, the mechanism by which reduced apoptosis leads to abnormal cap cell-cluster is still unclear. One possible explanation is that apoptosis of apical cells contributes to the migration of apical cells for appropriate shaping of cap cell cluster and determining the outline of germarium. We have now added these discussions in the revised manuscript as follows:

(Discussion, line 296-303)

On the other hand, the mechanism by which apoptosis of apical cells ensures the proper shaping of cap cell-cluster is still unclear. Notably, we found that suppression of apoptosis and misshaping of cap cell-cluster are correlated (Fig 4C-G, Fig 5D, E, and S10 Fig). It may be possible that apoptosis of apical cells contributes to appropriate migration of apical cells. Given that the migration of apical cells along the proximo-distal axis individualizes the cluster of terminal filament cells and forms the outline of each germalium unit (Fig. 1A) (12), the appropriate migration of cap cells would contribute to proper shaping of cap cell-cluster.

3. The conclusion that it is the apical cells that are relevant is not entirely clear given the complexity of the GAL4 patterns. For example, why is the RNAi effect with bab-1 stronger than c587 given that c587 is expressed more strongly in the apical cells adjacent to the terminal filaments? Images in S1 Fig are of wandering larval gonads. Since the RNAi effect was most pronounced in "before wandering" larvae (Fig 1F), images of larval gonads at those stages should be provided. These may provide additional insight into which specific cells are important.

Response:

We thank the reviewer for the comment. As pointed out by the reviewer, the strengths of Gal4 drivers, *C587-Gal4* and *bab1-Gal4*, and the strengths of resulting phenotypes may not be consistent. To test this, we analyzed the expression patterns of Gal4 drivers and found that the *C587-Gal4* is expressed only in part of the apical cells before wandering larvae while *bab1-Gal4* is expressed in almost all apical cells. This could be the reason why the phenotype by *C587-Gal4* was weaker than that by *bab1-Gal4*. We have now added these data in the revised manuscript as S3A-D Fig as follows:

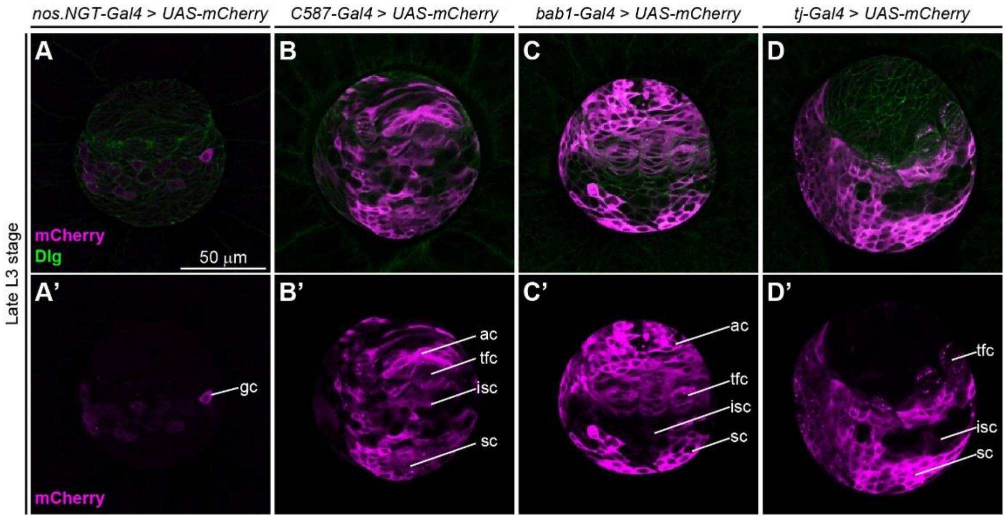

S3A-D Fig

(A-D) Female gonads of late L3 larvae (A-D) and wandering larvae (E-H) labeled with mCherry fluorescence (magenta) and anti-Dlg antibody (green).

(Results, line 128-131)

Unexpectedly, knockdown of *Ptp10D* or *sas* in germ cells using the *nos.NGT-Gal4* (S3A and E Fig) did not cause excessive GSCs (Fig 1E). Instead, knockdown of *Ptp10D* or *sas* using a somatic cell-specific *Gal4*-driver *C587-Gal4* (29, 30) (S3B and F Fig) caused significantly increased GSCs (Fig 1E).

(Results, line 133-138)

To clarify the responsible somatic cells that determine the number of GSCs, we further knocked down *Ptp10D* or *sas* using the *bab1-Gal4*, which drives gene expression at the larval stage in apical cells, terminal filament cells, and swarm cells, as well as at the adult stage in terminal filament cells and cap cells (31, 32) (S3C and G Fig) or the *tj-Gal4*, which acts at the larval stage in terminal filament cells, interstitial cells, and swarm cells, as well as at the adult stage in escort cells (32) (S3D and H Fig).

4. In Figure 2, it looks like most of the expression of Ptp10D is in the terminal filament cells, not apical cells as described. This needs to be explained. It is also intriguing that the Sas expression looks adjacent to the Ptp10D staining perhaps in the most basal of the apical cells.

Response:

We thank the reviewer for the comment. We carefully analyzed the expression pattern of Ptp10D and found that it is expressed in both apical cells and terminal filament cells (please see Fig. 1 for the reviewer (Fig. R1), below). As shown in Fig. R1, the mosaic analysis using *CoinFLP-Gal4>Ptp10D RNAi* revealed that the strong expression of Ptp10D at the boundary between apical cells and terminal filament cells was not suppressed by *Ptp10D*-RNAi in apical cells (yellow arrowheads) or terminal filament cells (green arrowheads) but was abolished by *Ptp10D*-RNAi in both cells (white arrowheads).

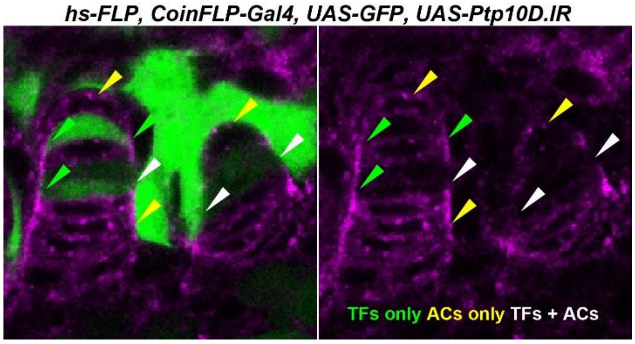

Fig. R1

(Left) Magnified image of female gonad in indicated genotype at wandering L3 stage is shown. Gonad was labeled with anti-Ptp10D (magenta) and GFP (green) (A and B). Coin-FLP induced flip-out clones in which *Ptp10D* was knocked down were induced by *hs-FLP* (37°C for 30 min,

1 days before wandering). (Right) Magenta channel of the left panel. Yellow arrowheads indicate boundaries between *Ptp10D*-RNAi apical cells and wild-type terminal filament cells. Green arrowheads indicate boundaries between wild-type apical cells and *Ptp10D*-RNAi terminal filament cells. White arrowheads indicate boundaries between *Ptp10D*-RNAi apical cells and *Ptp10D*-RNAi terminal filament cells.

We also agree with the reviewer that Sas is expressed more strongly in apical cells than terminal filament cells. We have now described these points in the legend of Fig 2 as follows:

(Fig 2-legend)

Ptp10D is expressed ubiquitously and exhibits strong expression at the boundary between apical cells and terminal filament cells. Sas::SGFP shows higher expression in apical cells, especially in the most-distal region, compared to other cell types at basal half of gonad.

5. Antibody staining on the Ptp10D mutant and knockdown larval gonads should be provided to demonstrate the specificity of the antibody.

Response:

Following the reviewer's suggestion, we examined the specificity of the antibody by staining *Ptp10D* mutant and knockdown larval gonads (please see below). We have now included these data in the revised manuscript as S7A-D Fig as follows:

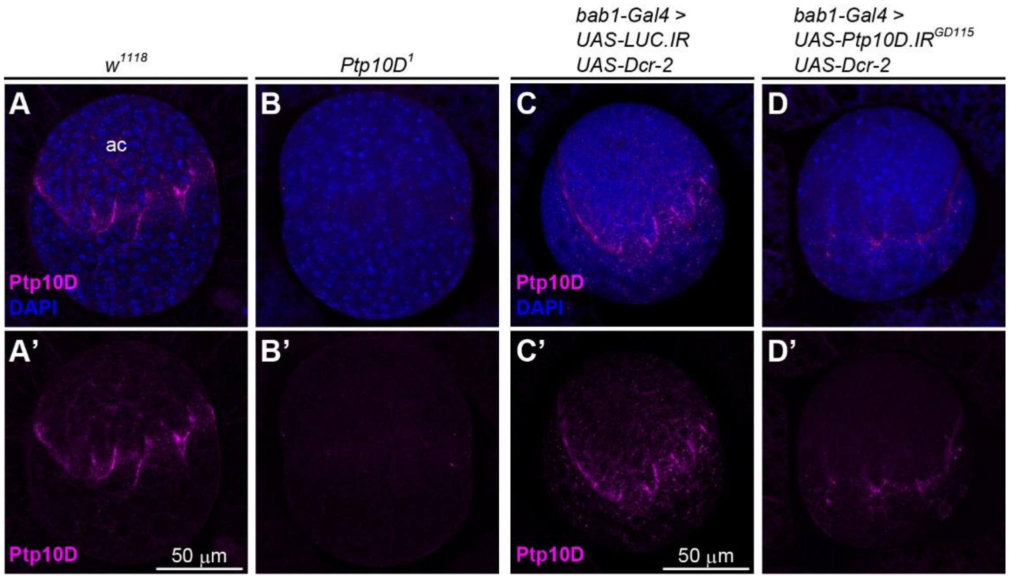

S7A-D Fig

(A-D) Female gonads of indicated genotypes (upper) at wondering L3 stages are labeled with DAPI (blue) and anti-Ptp10D antibody (magenta). The cell layer located at the opposite side of the fat body adherent surface is shown. Images are processed by Z-stack projection of two sections (corresponding to 2 µm thickness). (A'-D') Magenta channels of (A-D). Scale bars, 50 µm. *Ptp10D*^{1} exhibited a complete loss of Ptp10D in gonad (A and B). The knockdown of *Ptp10D* by *bab1-Gal4* reduced the Ptp10D staining especially in apical cells (C and D).

(Results, line 185-187)

We also confirmed that *Ptp10D*^{1} abolished Ptp10D protein expression in gonads and that *bab1*-Gal4-mediated knockdown of *Ptp10D* or *sas* reduces Ptp10D protein expression or Sas::SGFP in apical cells, respectively (S7 Fig).

6. In the apoptosis experiments, the effect in Figure 4C is quite modest, but the effect in D-G looks more convincing. It would be interesting to stain the p35 expressing flies for cDcp-1 or tunel as it is possible that p35 is not blocking the cell death effectively. In addition, blocking apoptosis with dronc RNAi or Diap1 might be more effective than the RHGmir and p35.

Response:

We thank the reviewer for the suggestion. Following the reviewer's comment, we performed anti-c-Dcp-1 staining of the gonad expressing *p35* or *RHG.miRNA*. Intriguingly, we found that *p35* and *RHG.miRNA* indeed did not strongly suppressed apoptosis. In addition, *Diap1* showed a stronger suppression of apoptosis than *p35* or *RHG.miRNA*. Therefore, we have added these data in the revised manuscript as Fig 4C and S10 Fig as follows:

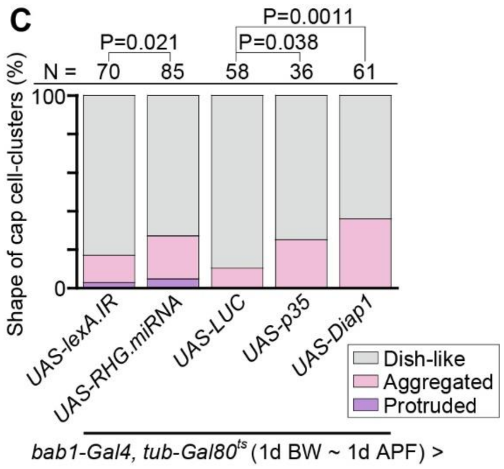

Fig 4C

(C) 100%-stacked bar graph represents % of germaria with normal (gray), aggregated (pink), and protruded (purple) cap cell-clusters in indicated genotypes.

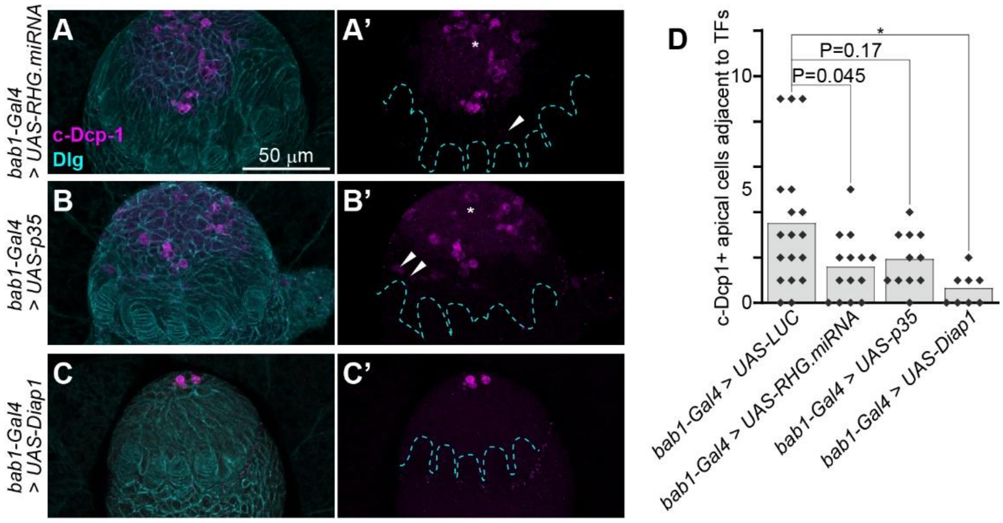

S10 Fig

(A-D) Female gonads at wondering L3 stage are labeled with anti-c-Dcp-1 antibody (magenta) and anti-Dlg antibody (cyan). The cell layer located at the opposite side of the fat body adherent surface are shown. Genotypes of samples are indicated at the left. Images are processed by Z-stack projection of two sections (corresponding to 3 μm thickness) to visualize the boundary between apical cells and terminal filament cells. (A'-C') Magenta channels of (A-C). Scale bars, 50 μm. Cyan dashed lines in (A'-C') indicate boundary between apical cells and terminal filament cells. Arrowheads in (A' and B') indicate the c-Dcp-1-positive apical cells which are adjacent to terminal filaments. Asterisks in A' and B' indicate accumulation of c-Dcp-1 positive cells at the proximal regions by unknown mechanism. (G) Bar graph overlaid with beeswarm plots represent numbers (No.) of c-Dcp-1-positive apical cells adjacent to terminal filaments (TFs) per larval gonads in indicated genotypes. *LUC* (*luciferase*) was used as control. P-values (*P < 0.01, 0.01 < "actual P-value") for Mann-Whitney U test are shown.

(Results, line 211-219)

To test this possibility, apoptosis was genetically blocked by overexpression of *RHG.miRNA*, an artificial microRNA that simultaneously suppresses *Drosophila* proapoptotic genes *reaper*, *head involution defective* (*hid*) and *grim* (36), baculovirus *p35*, an inhibitor of effector caspases (37), or *Death-associated inhibitor of apoptosis 1* (*Diap1*), an inhibitor of caspases (38). The stage-specific overexpression of *RHG.miRNA* (Fig 4B and C), *p35* (Fig 4C), or *Diap1* (Fig 4C) in gonadal apical cells by *bab1-Gal4* caused abnormal shape of the cap cell-cluster (Fig 4A, B and C) accompanied with increased GSCs (S8 Fig), which phenocopies loss of *Ptp10D* or *sas* (Fig 2E-G).

(Results, line 237-239)

We also confirmed that overexpression of *RHG.miRNA* or *Diap1* significantly reduced the number of c-Dcp-1-positive apical cells adjacent to the terminal filament cells, while overexpression of *p35* only showed a tendency to suppress apoptosis (S10 Fig).

Minor points

1. Fig 1 B,C would be helpful to show 3D image (movie) to make it easier to see all of the GSCs.

Response:

We thank the reviewer for the suggestion. We have now included 3D images (movies) of Fig 1 B, C as S1 Movie and S2 Movie in the revised manuscript.

2. They should provide numbers of GSCs in the apoptosis experiments (Figure 4) given how modest the effect was on cap shape.

Response:

Following the reviewer's suggestion, we quantified the number of GSCs when *p35*, *RHG.miRNA*, or *Diap1* was overexpressed. Consistent with the weak phenotypes in cap cell-cluster, the number of GSCs was moderately increased in these conditions. We have now added these data in the revised manuscript as S8 Fig as follows:

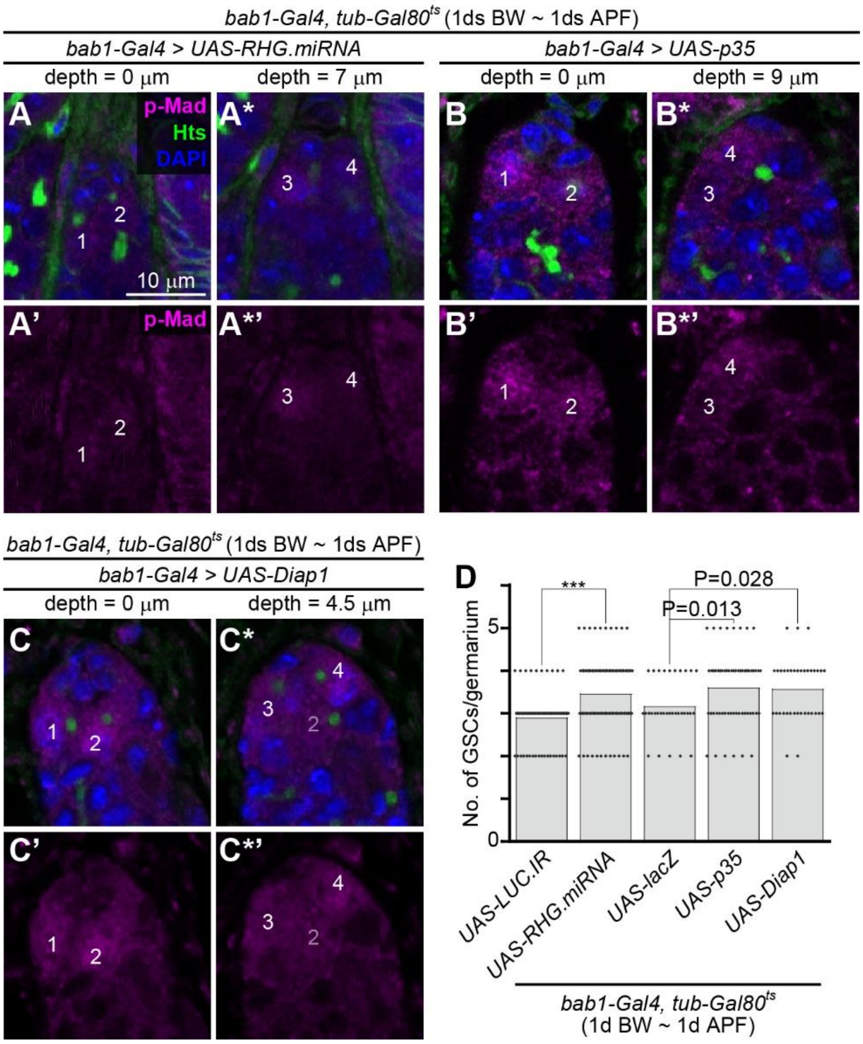

S8 Fig

(A-C) Proximal regions of virgin female germaria labeled with anti-p-Mad antibody (magenta), anti-Hts antibody (green), and DAPI (blue). Proximal is to the top. Germaria of indicated genotypes 2 days after eclosion that are treated with temperature-sift (29°C, 1d BW ~ 1d APF) are shown. Germaria with four GSCs are shown as examples. Numberings indicate residential GSCs. (A*-C*) Another optical section of (A-C). (A'-C', and A*'-C*') Magenta channels of (A-C and A*-C*). Scale bar in (A) is 10 µm, and applicable for (B, C, and A*-C*). (C) Bar graph overlaid with beeswarm plots represents numbers (No.) of GSCs per germarium in indicated genotypes. P-values (***P < 0.0001, *P<0.01, 0.01 < "actual P-value" < 0.05) for Mann-Whitney U test are shown.

(Results, line 215-219)

The stage-specific overexpression of *RHG.miRNA* (Fig 4B and C), *p35* (Fig 4C), or *Diap1* (Fig 4C) in gonadal apical cells by *bab1-Gal4* caused abnormal shape of the cap cell-

cluster (Fig 4A, B and C) accompanied with increased GSCs (S8 Fig), which phenocopies loss of *Ptp10D* or *sas* (Fig 2E-G).

(Results, line 239-243)

The moderate suppression of apoptosis by *RHG.miRNA*, *p35*, or *Diap1* was consistent with the moderate germarium phenotypes (Fig 4C and S8D Fig). These results support the notion that suppression of apoptosis in the larval gonad correlates with abnormalities in the adult germarium.

3. They should look at the EGFR reporter in the Ptp10D mutant background to confirm that it directly regulates EGFR in this context.

<u>Response:</u>

We agree with the reviewer that it is important to examine ERK activity in *Ptp10D* mutants. Following the reviewer's comment, we investigated ERK activity using anti-phosphorylated ERK (pERK) antibody and found that ERK activity was indeed elevated in the apical cells of *Ptp10D*^{1} mutants. We have now added these data in the revised manuscript as Fig. 3 A-C as follows:

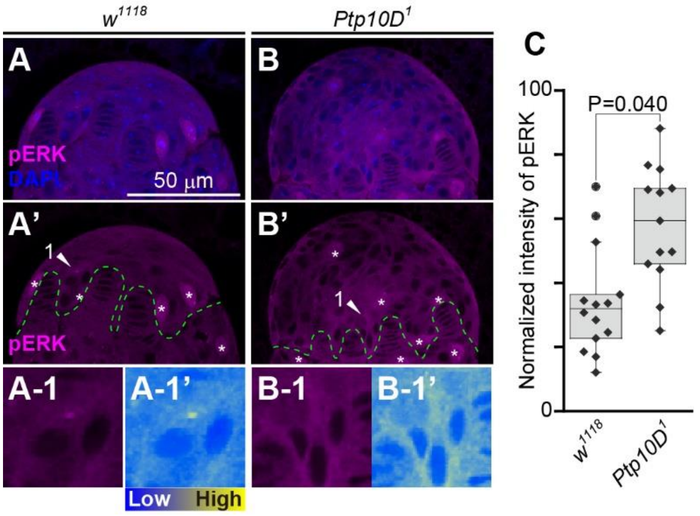

Fig 3A-C

(A and B) Female gonads of late wandering larvae in *w*^{1118} and *Ptp10D*^{1} homozygote were labelled with anti-pERK antibody (magenta) and DAPI (blue). (A' and B') Magenta channels of (A and B). Asterisks in (A' and B') indicate the non-specific staining of anti-pERK antibody in mitotic cells. Green dashed lines in (A' and B') indicate the boundaries between apical cells

and terminal filament cells. Apical cells indicated by arrowheads 1 in (A' and B) are shown in (A-1 and B-1). Heatmap images of (A-1 and B-1) are shown in (A-1' and B-1'). (C) Boxplot overlaid with beeswarm plot represents the normalized intensity of pERK staining. Data values from *w*^{1118} and *Ptp10D*^{1} are plotted. P-value for Mann-Whitney U test are shown.

(Results, line 194-197)

Indeed, *Ptp10D*^{1} exhibited elevated Egfr signaling in gonadal apical cells, as visualized by anti-phospho-ERK (pERK) staining (Fig 3A-C), and stage-specific knockdown of *Egfr* in gonadal apical cells by *bab1-Gal4* significantly suppressed the abnormal shape of the cap cell-cluster in *Ptp10D*^{1} (Fig 3D).

4. The puc overlap with Dcp-1 is not convincing in Figure S6.

Response:

We thank the reviewer for the comment. We have now replaced the image with a better one in S12E Fig (please see below).

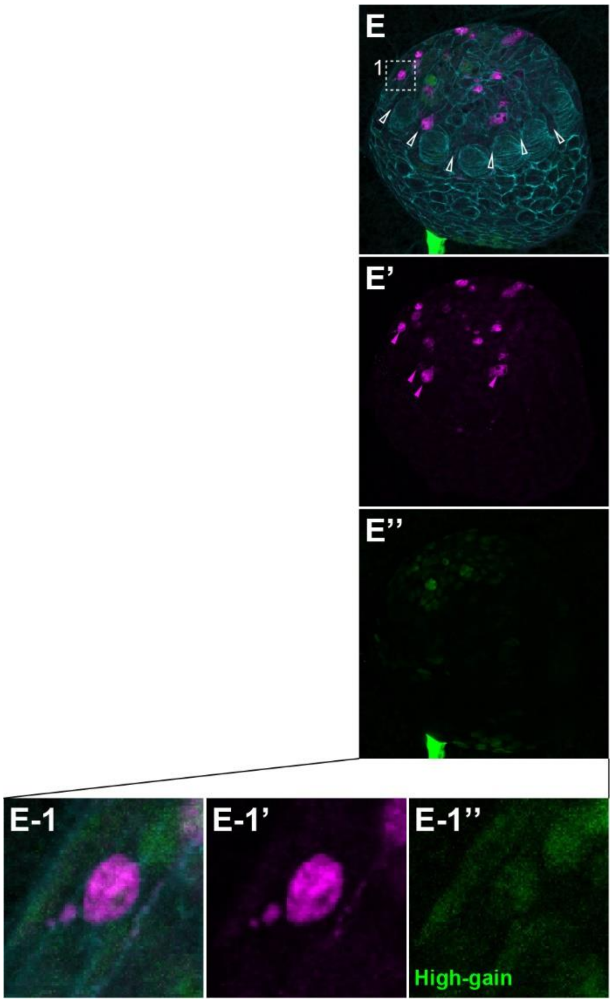

S12 Fig

(E-1-E-1'') High-magnification images of white-dashed box 1 shown in E. Gains of green channels are amplified to enhance the signal. Some apical cells adjacent to terminal filament cells show *puc*-Stinger signal (E-1'') and undergo apoptosis (E-1').

5. Discussion – the ratio of germ cells to follicle cells might cause more disruptions to oogenesis. This would be seen as increased apoptosis of germcell clusters in the germarium. Were any defects seen?

Response:

Following the reviewer's comment, we analyzed apoptosis of germ cell clusters. As a result, we did not observe significant increase in the number of c-Dcp1 positive cells in

germ cells of germarium or oocytes in *Ptp10D*[1] mutants. We have now added these data in the revised manuscript as S14 Fig as follows:

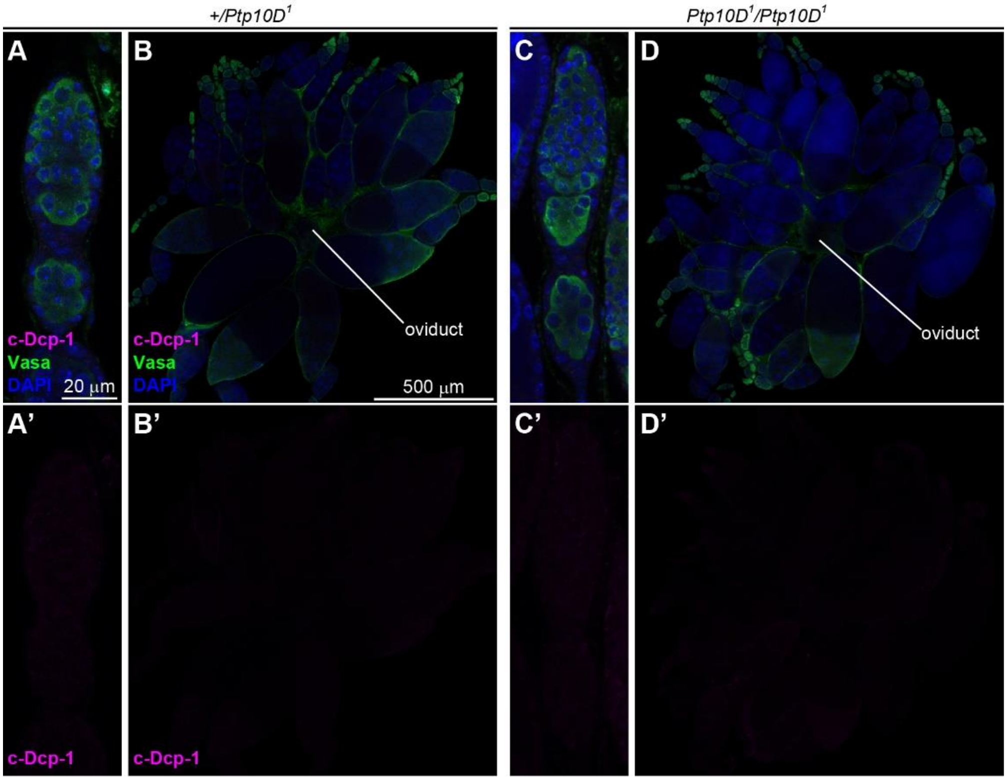

S14 Fig

(A-D) Virgin female germaria (A and C) and ovaries (B and D) loosened radially 1 day after eclosion are labeled with anti-c-Dcp-1 antibody (magenta), anti-Vasa antibody (green), and DAPI (blue). Control *Ptp10D*[1] heterozygote (A and B) and *Ptp10D*[1] homozygote (C and D) are shown. Scale bars, 20 µm (A and C) or 500 µm (B and D). *Ptp10D*[1] homozygote did not exhibit increase in the number of apoptotic cells in germarium (C) or oocytes (D).

(Results, line 279-280)

Ptp10D[1] females did not show increased apoptosis in germ cells during oogenesis in germarium or oocytes (S14 Fig).

(Discussion, line 319-321)

We also did not find any increase in the number of apoptotic cells as a defect in oogenesis in *Ptp10D* mutants (S14 Fig).
